# The Evaluation of Drug Delivery Nanocarrier Development and Pharmacological Briefing for Metabolic-Associated Fatty Liver Disease (MAFLD): An Update

**DOI:** 10.3390/ph14030215

**Published:** 2021-03-04

**Authors:** Reem Abou Assi, Ibrahim M. Abdulbaqi, Chan Siok Yee

**Affiliations:** 1 Thoughts Formulation Laboratory, Discipline of Pharmaceutical Technology, School of Pharmaceutical Sciences, University Sains Malaysia, Minden 11800, Penang, Malaysia; reembouassi@student.usm.my; 2 Discipline of Pharmaceutical Technology, College of Pharmacy, Al-Kitab University, Altun-Kupri, Kirkuk 36001, Iraq; ibrahim.m.abdulbaqi@uoalkitab.edu.iq; 3 Pharmaceutical Design and Simulation (PhDS) Lab, Discipline of Pharmaceutical Technology, School of Pharmaceutical Sciences, University Sains Malaysia, Minden 11800, Penang, Malaysia

**Keywords:** non-alcoholic fatty liver disease (NAFLD), metabolic fatty liver disease (MAFLD), insulin resistance, obesity, nanoformulations, nanotechnology, nanocarrier, nanosystem

## Abstract

Current research indicates that the next silent epidemic will be linked to chronic liver diseases, specifically non-alcoholic fatty liver disease (NAFLD), which was renamed as metabolic-associated fatty liver disease (MAFLD) in 2020. Globally, MAFLD mortality is on the rise. The etiology of MAFLD is multifactorial and still incompletely understood, but includes the accumulation of intrahepatic lipids, alterations in energy metabolism, insulin resistance, and inflammatory processes. The available MAFLD treatment, therefore, relies on improving the patient’s lifestyle and multidisciplinary pharmacotherapeutic options, whereas the option of surgery is useless without managing the comorbidities of the MAFLD. Nanotechnology is an emerging approach addressing MAFLD, where nanoformulations are suggested to improve the safety and physicochemical properties of conventional drugs/herbal medicines, physical, chemical, and physiological stability, and liver-targeting properties. A wide variety of liver nanosystems were constructed and delivered to the liver, only those that addressed the MAFLD were discussed in this review in terms of the nanocarrier classes, particle size, shape, zeta potential and offered dissolution rate(s), the suitable preparation method(s), excipients (with synergistic effects), and the suitable drug/compound for loading. The advantages and challenges of each nanocarrier and the focus on potential promising perspectives in the production of MAFLD nanomedicine were also highlighted.

## 1. From NAFLD to MAFLD—What Is Going on?

Non-alcoholic fatty liver disease (NAFLD) is defined by the presence of excessive fat in the liver, which is identified either by imaging or liver biopsy. It is the term used to describe various histologic anomalies, ranging from benign steatosis to non-alcoholic steatohepatitis, in people who consume little to no alcohol. NAFLD has a substantial potential to progress to cirrhosis, hepatocellular carcinoma, end-stage liver disease, liver-related death, and recurrence after transplantation [1]. In 2013, scientists released an alarm about the future of liver diseases in which NAFLD is turning to become an epidemic [2]. Given the drastic and growing prevalence of NAFLDs, which affect more than one-quarter of the world’s population [3,4], the severe hepatic and extra-hepatic sequelae, and the inadequacy of treatment options, thus, re-naming and re-defining the disease are welcome [5]. The term “fatty liver” was first described in 1836, while the name “NAFLD” was first used in 1980 and for 40 years until 2020 when it was re-evaluated for a more precise nomenclature indicating the existence of metabolic dysfunction, rather than the absence of other conditions, such as alcohol intake, as a safe limit of alcohol consumption could not be set [6]. As a result, the well-known NAFLD nomenclature was replaced by metabolic-associated fatty liver disease (MAFLD) [7,8,9], to open the door to the development and implementation of a set of “positive” criteria for defining the condition rather than depending on a “non” or “negative” definition [5].

MAFLD is expected to overburden the annual economy dependent on Medicare recipients with an increase of USD 188 billion in corporate costs alone. Approximately the same costs are split into four European countries, including Germany, France, Italy, and the United Kingdom, with the highest costs for patients aged between 45 and 65 [10], with no apparent gender preference as some research is suggesting that MAFLD is more common in females [11,12,13,14,15] while others were also highlighting that it is more common in males [16,17,18,19,20], this conflict could be due to the limited data about MAFLD in females rather than males, as will be noticed in the illustrated in vivo models later in this article. Interestingly, MAFLD impacts children’s health in an asymptomatic profile that is incidentally diagnosed at a mean age of 11–13 years [21,22,23]. Overall, MAFLD mortality is escalating [24] with an estimated rise between 65% and 100% from 2019 to 2030 in the Asia-Pacific region alone [25], as well as the US, China, and France, which is combined with an alarming MAFLD predicted prevalence escalation to occur in Germany, Italy and the UK by 2030 [26]. 

Clinically, MAFLD is considered to be the hepatic manifestation of multiple metabolic syndromes, which is present when three or more of the following conditions are met, including obesity, elevated glucose, blood pressure, triglyceride, as well as high-density lipoprotein cholesterol [27]. To recapitulate MAFLD pathogenesis’s complexity, “multiple hit theory” was recently established, where multiple synergistically acting factors are involved in the disease incidence and progression. These factors include genetic predisposition, dietary factors, altered gut microbiota and insulin-resistance-induced alteration in production and secretion of adipokines, mitochondrial dysfunction, and endoplasmic reticulum stress. These factors contribute to the development of steatosis and steatohepatitis and reflect different disease patterns among MAFLD patients [28].

## 2. Available MAFLD Treatments and Their Efficacy

MAFLD’s etiology is multifactorial and still incompletely understood, but involves accumulation of intrahepatic lipids, alterations of energy metabolism, insulin resistance, and inflammatory processes [29]. Consequently, the available MAFLD therapy relies on changing the patient’s lifestyle and multidisciplinary pharmacotherapeutic options.

### 2.1. Non-Pharmacological Approach

In the absence of approved single pharmacological therapies for MAFLD, the current European Association for the Study of the Liver, European Association for the Study of Diabetes, and European Association for the Study of Obesity Clinical Practice Guidelines for the management of MAFLD recommend lifestyle modification as the strategy of choice for prevention and improvement [30]. Diet calorie reduction is the first therapeutic technique in MAFLD; the diet should minimize both saturated fat intake to <7% of the total calories and trans-fat intake, and maintain dietary cholesterol intake at <200 mg/day, and total fat at 25% to 35% of the total calories in order to achieve moderate weight loss (7 to 10%) [31]. In addition, increased exercise, such as 150 to 200 min/week of moderate-intensity aerobic physical activity is reported to enhance liver histology, insulin resistance, and life quality. Therefore, this should form the basis of any treatment strategy [30,32,33]. This approach may be challenging to maintain over the long-term, even with the implementation of cognitive-behavioral therapy; thus, the pharmacological intervention will be the other choice in such cases.

### 2.2. Pharmacological Approach

#### 2.2.1. Anti-Obesity Drugs

The addition of pharmacotherapy resulted in more significant weight loss and weight loss maintenance than lifestyle modifications alone. Currently, there are nine pharmacological interventions approved by the US Food and Drug Administration (USFDA) that can be used for weight loss, including phentermine, diethylpropion, benzphetamine, phendimetrazine, orlistat, phentermine/topiramate extended release, lorcaserin, bupropion/naltrexone, and liraglutide [34]. In MAFLD, anti-obesity medications did not directly affect the liver independent of the effect on weight loss. For now, orlistat and sibutramine are mainly the available options for the long-term prescription. However, there are still insufficient safety data regarding the long-term outcomes of anti-obesity therapy [35]. The choice to initiate obesity drug therapy should be controlled, first by avoiding contraindications and drug–drug interactions, and second, by choosing a therapy with a mechanism of action that targets the patient’s dietary behavior; close monitoring is vital for assessing efficacy, tolerability, and switching to an alternative option if needed [36].

#### 2.2.2. Hypoglycemic Agents

Since MAFLD and type 2 diabetes mellitus share pathophysiological characteristics, such as insulin resistance, hypoglycemic agents have used mainly metformin and thiazolidinedione [37]. In the case of metformin administration (0.5 to 3 g/day), a meta-analysis reported its use to be essential for improving liver function and body composition in non-diabetic MAFLD patients [38,39]. However, some increase in insulin sensitivity disappears after three months of metformin use in MAFLD patients [40,41], with no assessed direct impact of metformin on the MAFLD activity score [42]; side effects include gastrointestinal tract (GIT) discomfort and general weakness with records of interactions when used with various medications. Although the use of thiazolidinediones in MAFLD is reported at a much lower dose (4 to 45 mg/day) compared to metformin and is a promising choice in the treatment of MAFLD manifestations due to its beneficial impact on insulin resistance and hepatocyte fatty acid metabolism, the side effects (lower extremity edema, and weight gain) and the need for long-term use establish a disadvantage [43,44]. Scientists recommend further investigations on the use of recent hypoglycemic agents, including glucagon-like peptide 1 receptor agonists, dipeptidyl peptidase 4 inhibitors, and sodium/glucose co-transporter 2 inhibitors and their impact on MAFLD—in particular, the oral versus parenteral route of administration profile in humans [37,45,46].

#### 2.2.3. Lipid-Lowering Agents

Statins, fibrates, and omega-3 polyunsaturated fatty acids are commonly used to manage dyslipidemia. Due to their potential antioxidant properties and favorable effect on adiponectin levels, they are suggested to benefit MAFLD patients [47]. Statins function as anti-MAFLD by controlling metabolic risk factors with dose-related hepatotoxicity [48]; higher lipophilic statins show a greater hepatic excretion rate, while lower lipophilic statins exhibit more pronounced active renal excretion [49,50]. Food intake affects the bioavailability of statins, as most of them have low bioavailability, except for Pitavastatin (>80%), while several meta-analyses of major statins have consistently recorded modestly increased risk of incident diabetes (9–13%) in MAFLD patients [48,51,52,53]. In addition, as per a pilot study, other options such as fibrates are considered safe and improve metabolic syndrome, glucose, and liver tests; although their effects on liver histology are minimal [54], fibrates are suggested to be useful for the prevention and management of MAFLD [55]. Other lipid-lowering agents, such as ezetimibe, are reported to attenuate hepatic steatosis and may benefit MAFLD biochemical markers (i.e., fatty acids). The majority of the results in such regard are from animal studies, which not are always compatible with human physiology, as authors point out [56]. Indeed, the study on humans suggested the possibility of the animal results heterogenicity, where later ezetimibe was reported to improved hepatic fibrosis in 80 patients’ results, but increased hepatic long-chain fatty acids [57].

#### 2.2.4. Cytoprotective and Antioxidant Agents

Inflammation and oxidative stress are believed to play a role in the pathogenesis of MAFLD, where anti-inflammatory and potent antioxidants own a crucial role in anti-MAFLD therapy, such as bile acids (ursodeoxycholic acid) [58,59]. In such regard, phytochemicals and micronutrients are also used, including flavonoids [60], polyphenols [61], carotenoids [62], and phenolic compounds [63], as well as vitamin E [64], and silymarin [65]. They act as attractive therapeutic agents for short-term treatment until the full profile of safety and effectiveness is further explored with their impact specifically in diabetic and non-diabetic MAFLD cases [46,66].

### 2.3. Surgical Intervention

With a causal link established between MAFLD and obesity-related metabolic syndrome, bariatric surgery interventions promote weight loss and would be expected to improve MAFLD; the surgery is a debated option for MAFLD treatment where, despite it having the potential for inducing excellent weight loss and possibly improving the symptoms of metabolic syndrome, type 2 diabetes mellites, and potentially reversing the pathological liver changes in MAFLD patients [34,67], it could also, reportedly, be a secondary outcome of the bariatric surgery [68], while other patients developed new or worsened features of MAFLD, including the development de novo, worsening of fibrosis, MAFLD activity score, MAFLD histology, and change in liver volume [69]. Performing this costly surgery might add a few years to the patient’s life, but it does not reduce the overall healthcare costs in the long-term [70]. On the other hand, liver transplantation cures cirrhosis but does not treat MAFLD’s underlying metabolic disease. Thus, strategies to control comorbidities in patients with MAFLD before transplant are needed to decrease waitlist mortality, and the recurrence of MAFLD after liver transplant [71].

## 3. Nanomedicine Alternatives for MAFLD Smart Drug Delivery

**Figure 1:**
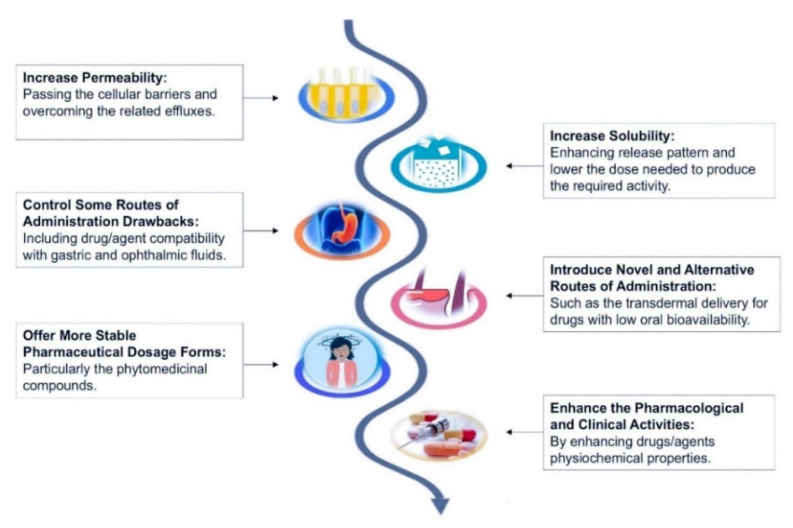
The main challenges that nanoformulations are overcoming to enhance drug/phytochemical delivery.

Other currently emerging approaches to MAFLD therapy include developing new targeting drugs that are already under phase 2 and phase 3 trials [72], or improving the safety and physicochemical properties of conventional drugs and herbal medicines, as well as physical, chemical, and physiological stability, along with liver-targeting properties using a wide range of nanoformulation techniques [73], as illustrated in Figure 1. The United States National Nanotechnology Initiative described nanotechnology as science, engineering, and nanoscale technology, approximately 1 to 100 nanometers (nm) [74], however, several formulations were reported under the nanosystem umbrella for the merit of providing a particle size scale below 1000 nm.

**Figure 2:**
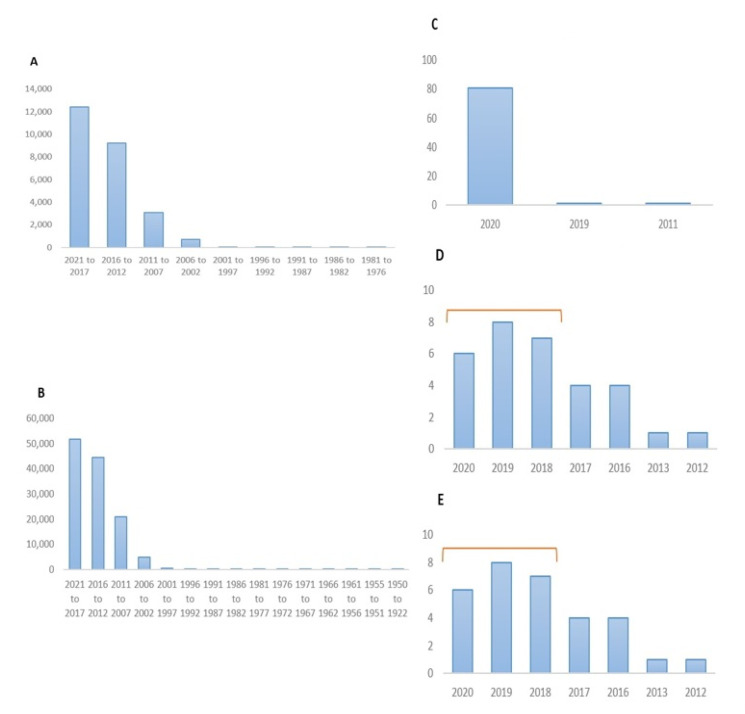
The published article number from the PubMed search (October 2020) vs. the years, using the keywords of **A**: NAFLD, **B**; Nano, **C**: MAFLD, **D**: NAFLD nano, **E**: MAFLD nano with a peak in research since 2018 (expressed within the orange line). NAFLD, non-alcoholic fatty liver disease; MAFLD, metabolic fatty liver disease.

Compared to the research size conducted on MAFLD alone, a minimal number of nanoformulations directly address this disease by evaluating the designed nanosystems on the in vitro, ex vivo and in vivo models. Searching the PubMed database alone showed around 122,877 articles covering “nano” topics, out of which only 31 were in the MAFLD field, which started to trend in 2018. Using the searching keyword Nano-MAFLD or Nano-NAFLD yielded the same article number, while searching NAFLD or MAFLD alone yielded 25,471 and 83 studies, respectively (October 2020), as illustrated in Figure 2.

## 4. Opportunities and Limitations of Nanosystems for MAFLD Therapy

### 4.1. Nanoparticles

#### 4.1.1. Polymeric Nanoparticles

**Figure 3:**
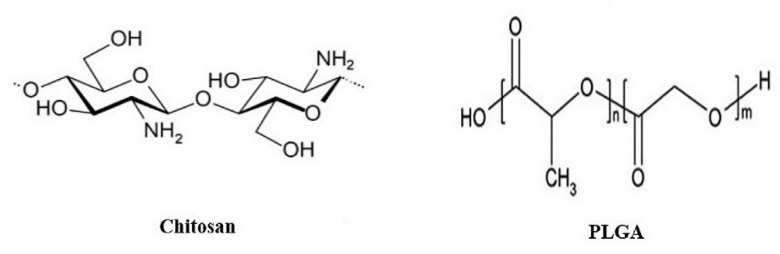
Chemical structures of the most used polymers in engineering polymer-based nanoparticles for MAFLD therapy.

Polymer nanoparticles (P-NPs) are the most widely used nanocarriers in the pharmaceutical sector to develop controlled, sustained and burst drug delivery systems with release profiles directly related to the constructed polymeric shell. P-NPs can be either nanospheres or nanocapsules, with particle size (PS) ranging from 1 to 1000 nm and two fundamental preparation techniques, including “top-down” and “bottom-up” methods [75,76,77]. They can be used to mask the taste and to protect drug/compounds from environmental and gastric degradation, offering enhanced bioavailability, increased stability of any volatile materials with non-immunogenicity, and a low to nontoxicity nature [78,79]. Challenges of this nanosystem design are merely summed up by the proper selection of the polymer [80]. Within the P-NP design for MAFLD therapy, polymer use is mainly limited to chitosan, and poly lactic-glycolic acid (PLGA) (Figure 3), with promising results of chitosan alone as an excipient owning synergetic anti-MAFLD effects. Other polymers with anti-MAFLD therapy that are not used in P-NP formulation to address the disease include accase-catalyzed catechin polymers [81], pyrrole-based polymers [82], Hericium Erinaceus exo-polymers [83], beta-cyclodextrin [84,85], and colestipol [86].

These polymers were reported to have a lowering influence of cholesterol, low-density lipoproteins (LDL)-cholesterol fraction, hyperlipidemia, cholesterol and lipid levels, respectively. Additionally, there is a need to investigate glucose-lowering polymer synergetic effects, such as cellulose, which positively impacts diabetes mellitus type 2 [87,88,89], and inulin, which was reported to reduced serum insulin levels but simultaneously increased body weight in diabetic rats [90]. Thus, preliminary studies are recommended when exploring glucose-polymers as excipients for nanoparticles, such as dextrans, icodextrin, and amylose, to ensure that there are no short-term or long-term consequences on MAFLD patients, including the blood glucose levels [91,92,93,94].

Chitosan is a golden approach for MAFLD polymeric drug delivery, which is not fully explored yet. It is highly available as a natural cationic aminopolysaccharide polymer of marine origin, prepared from crustaceans’ shells, and classified as generally recognized as safe (GRAS) by USFDA [95]; thus, it owns ideal biological properties. It also exhibits hepato-protective effect [96], regulatory impact of carbohydrates and lipid metabolism, ameliorating insulin resistance, and decreasing the prevalence of MAFLD [97,98], along with the decrease in aspartate aminotransferase (AST) and alanine aminotransferase (ALT) serum levels in the in vivo studies [99]. In addition, it owns a lipid-lowering capacity, through promoting weight loss, lowering LDL, and boosting high-density lipoprotein (HDL), with a suggested mechanism linked to the polymer’s positive charge, which attracts the negatively charged fatty acids and bile acids binding them to the indigestible chitosan fiber [100]. Interestingly, this unique polymer can deliver genes and target the liver [101,102,103], which nominates it to be the polymer of choice for smart anti-MAFLD nanomedicine delivery.

In MAFLD patients, a decline in nicotinamide adenine dinucleotide (NAD1) biosynthesis is observed; thus, a high dose of nicotinamide (NAD1 precursor) with a dose of the relatively unstable antioxidant, ascorbic acid, was usually administrated. Accordingly, chitosan nanoparticles were prepared by the ionotropic gelation technique to overcome the drawbacks of high dose intake and instability challenges. On the in vivo level (using male albino rats), these NPs showed an insulin-resistant status amelioration, and reduction in ALT, AST, as well as liver tissue total cholesterol, triglycerides, and 8-hydroxy-2-deoxyguanosine (8-OHdG) levels [104]. The NPs also decreased oxidative and nitrosative stresses along with a significant increase in the hepatocellular energy upon the oral intake of only 10 and 20 mg/kg of loaded nicotinamide and ascorbic acid in NPs in comparison to their conventional forms oral dose of 100 mg/kg. Both chitosan nanoparticles were suggested to be cytocompatible, with encapsulation efficiency (EE%) of 75% and 85.5%, respectively, PS less than 200 nm and a positive zeta potential (ZP) (22.5 to 29.8 mV). Authors further suggest that the positive charge of these NPs, and the use of chitosan, would offer superior stability and a controlled-release manner, respectively, of the loaded materials. 

Poly lactic-glycolic acid (PLGA) is another polymer known to be a biodegradable, highly biocompatible polymer from the family of USFDA-approved polymers, with the ability to deliver active ingredients, proteins, and macromolecules [105], and promote their transportation across biological barriers. Mostly it is used for the intravenous route of administration (IV) with an accelerating potential for oral drug delivery during the last decade [106]. To the best of our knowledge, there is no study about the synergetic impact of PLGA alone on one or more of MAFLD manifestations.

In a first-time report, PLGA-based curcumin NPs were evaluated clinically in a double-blind, randomized, placebo-controlled study on 84 overweight/obese MAFLD patients. Curcumin is considered a folk medicine in MAFLD therapy primarily because of its antioxidant and anti-inflammatory properties. Treating patients between the age of 25 and 50 with supplementation of curcumin PLGA-NPs at 40 mg capsules/day after meals for three months leads to improved glucose, lipids, inflammation, and nesfatin, hepatic transaminases, and fatty liver degree indices [107,108]. These curcumin PLGA-NPs were initially patented turmeric-based colloidal dispersion technology named THERACURMIN, with a limited pharmaceutical characterization of PS 0.19 µm and stability of 28 days in comparison to 1 h for pure curcumin powder. The THERACURMIN was found to have the ability to raise the plasma concentration of curcumin by 39.8 to 81.7 times at 1–2 h after oral administration, compared with pure curcumin powder, yielding a higher bioavailability of curcumin by 40 folds [109]. Further trials of curcumin loaded in other nanocarriers as anti-MAFLD prospective remedies are encouraged to provide a better clinical and pharmacological understanding through expanding the studied variables, mainly ethnicity, age, group number and the preparation recipe with full characterization and evaluation. 

Another phytomedicine, resveratrol, from the polyphenol group, has been identified as a potential new pharmacological approach in the treatment of MAFLD [110]; however, its low solubility, could govern its effects that may be improved through encapsulating it with freeze-dried PLGA-NPs by the evaporation method. This has also improved its stability (up to 6 months under 4 °C) and bioactivity. The resulting resveratrol PLGA-NPs had PS of 176.1 nm with Đ of 0.152 and ZP of −22.6 mV, while the EE% and loading percentage were 97.25% and 14.9%, respectively. The NPs offered a sustained release profile of the resveratrol with a much lower release in the acidic medium than the alkaline one. Resveratrol PLGA-NPs was more effective in alleviating lipogenesis, promoting lipolysis, and reducing hepatocellular proliferation compared to the free resveratrol when studied on the fat-emulsion-treated human hepatic cells (HepG2), which were incubated with oleic acid for 24 h to induce steatosis and build a closer cell culture model of MAFLD [111]. In this work, authors recommended further in vivo studies on such NPs to correlate in vitro and in vivo results.

On the nano-molecular level, a novel polymer, lactosylated poly(2-(dimethylamino) ethyl methacrylate, was used to construct NPs and load it with “miR-146b mimic”, which plays a vital role in the regulation of metabolic gene expression and MAFLD therapy through its ability to modulate hepatic lipid homeostasis. The resultant NPs had PS, Đ, and a positive ZP of 150 to 350 nm, 1.21, and 10.3 mV, respectively. The loaded formulation was cytocompatible and efficiently capable of directly targeting the hepatocyte and has been taken up by HepG2 and mouse hepatocyte cell lines (AML12). In addition, these NPs showed the ability to significantly suppress the formation of lipid droplets on the in vivo level, offering a specific liver delivery, with a significant inhibition to the lipid accumulation when injected via female C57BL/6 mice tail vein in a single dose of 1 mg/kg [112]. 

#### 4.1.2. Metal-Oxide-Based Nanoparticles

The metallic NPs (M-NPs) consist of a metallic core with a standard PS of 10–200 nm. They can comprise pure metallic substances (e.g., gold, silver, copper, platinum, and palladium) or metallic oxides (e.g., zinc oxide, titanium dioxide, cerium oxide, iron oxide), while their comparatively small PS is responsible for their high surface area to material ratio, leading to disproportionately more considerable exposure to the body [113]. Size and shape are the two major factors responsible for M-NPs’ properties robustness, any variation in them is significantly reflected in the M-NPs’ color, melting temperature, and electrical conductivity, even if the two NPs are made of the same pure metal [114]. They are known for their strong plasma absorption offering controlled release pattern, in addition to their applications in biomedical sciences including biological system imaging and disease diagnosis, with the possibility of large-scale production [115,116]. M-NPs can be synthesized in different shapes, including spheres, rods, and tubes [117]; indeed, their instability, challenging synthesis with the presence of impurities, and toxicity are matters of controversy [115,118]. 

**Figure 4:**
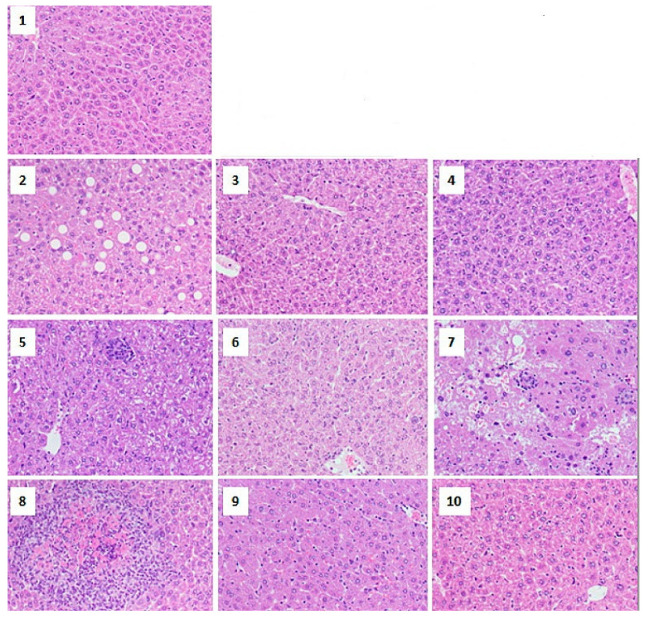
Histopathology of the liver after consuming 100 µL gold nanoparticles by different groups of Swiss albino female mice via intraperitoneal injection at different periods and particle size (PS). (**1**) Control mouse liver section showing normal hepatic architecture, (**2**) marked steatosis, and the abundance of micro and macro vesicles after one-day consumption at 5 nm, (**3**) and (**4**) look healthy with normal hepatocytes after one-day consumption at 20 nm and 50 nm, respectively, (**5**) cytoplasmic degeneration and some aggregation of inflammatory cells after seven days consumption at 5 nm, (**6**) looks healthy with mild activation of Kupffer cells after seven days consumption at 20 nm, (**7**) multi-necrotic foci filled with hemorrhage and also the presence of infiltrative cells after seven days consumption at 50 nm, (**8**) necrotic foci filled with edema and surrounded by inflammatory cells after one and seven days consumption at 5 nm, (**9**) and (**10**) look healthy with bi-nucleated cells and the activation of Kupffer cells after one and seven days consumption at 20 nm and 50 nm, respectively. Adapted from reference [120].

In particular, M-NPs’ toxicity is of concern, as the liver is the first point of contact for NPs entering the circulatory system; an example of such toxicity histological observation is illustrated in Figure 4. In this regard, NPs that can escape pre-systemic elimination could eventually accumulate in the liver post-entry, leading to profound interactions with hepatic cells and other non-parenchymal cells. Given the fact that M-NPs are notoriously resistant to degradation, this could lead to long-term persistence and subsequent hepatic toxicity [113]. In contrast, recent studies are reporting these NP benefits in healing the liver from critical situations, including the titanium dioxide (TiO_2_) and silicon dioxide (SiO_2_) NPs through inhibiting the cellular hepatic fibrosis [119], with no data on the possibilities of owning anti-MAFLD activities.

Interestingly, the spherical cerium dioxide (CeO_2_) NPs with PS of 4–20 nm paves the way in anti-MAFLD therapy through enabling the reduction in hepatocyte lipid droplets size and content, the hepatic concentration of triglyceride, and cholesterol ester-derived fatty acids, as well as the expression of several genes involved in cytokine, adipokine, and chemokine signaling pathways [121]. These nanoparticles’ original formulator suggested their hepatoprotective and therapeutic value in chronic liver disease [122]. Later, they were reported to be up-taken by HepG2 cells line that were intentionally treated with oleic and palmitic acids to induce the hepatosteatotic condition. These NPs have also reduced oxidative stress, improved cell viability, and reduced fatty acid content in steatotic conditions by inducing specific changes in fatty acid metabolism, suggesting their potential for treating MAFLD [123]. 

#### 4.1.3. Nanographene Oxide Particles

**Figure 5:**
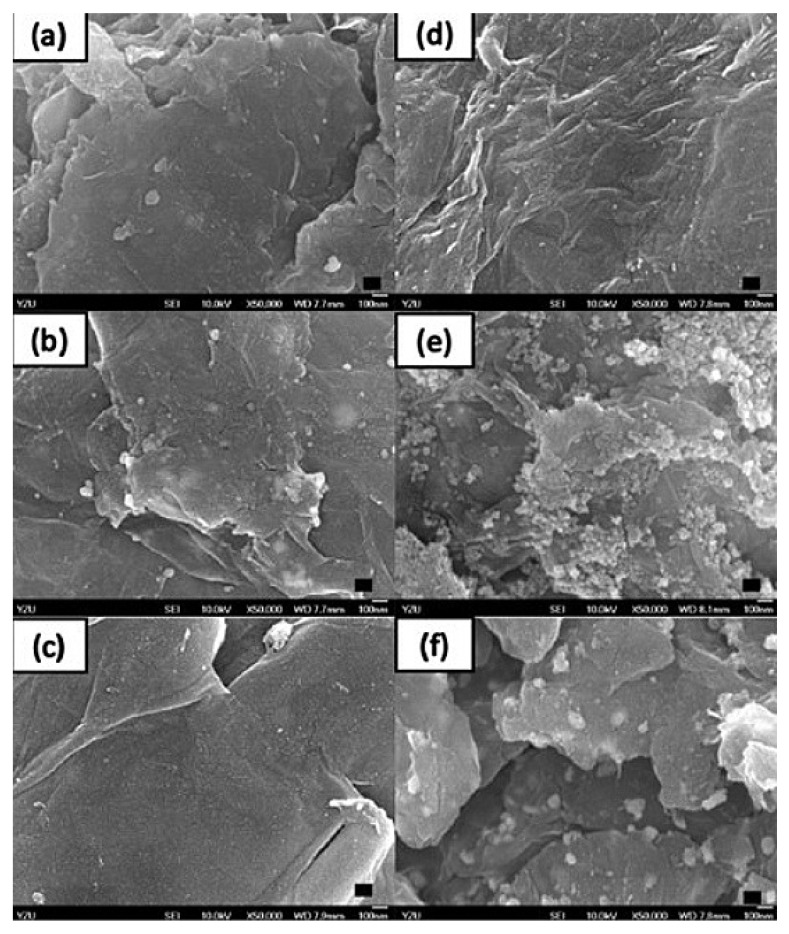
Scanning electron microscopy images of (**a**) 5.34% silver nanoparticle-embedded graphene oxide (Ag/rGO), (**b**) 7.49% Ag/rGO, (**c**) 6.85% zinc oxide nanoparticle-embedded graphene oxide (ZnO/rGO), (**d**) 16.45% ZnO/rGO, (**e**) 3.47% silver nanoparticle, and 34.91% zinc oxide nanoparticle-embedded graphene oxide (Ag/ZnO/rGO), and (**f**) 7.08% silver nanoparticle and 15.28% zinc oxide nanoparticle-embedded graphene oxide (Ag/ZnO/rGO). Adapted from reference [142].

Graphene was first reported for drug delivery in 2008, with an earlier established employment in the fields of photonics and electronics [124,125], while in nanomedicine it is considered the future boon of biotechnological kits and drug delivery for theranostics, high throughput biosensors and bioassay, smart scaffolds for tissue regeneration, and ultra-high sensitive biomarkers [126,127,128]. These various applications could be due to their wide PS range in both nano- and microscales [129,130]. Nanographenes (NGs) offer a controlled and slow-release profile [131,132], while their synthesis using the “bottom-up” method is considered more appropriate than the “top-down” one in terms of the produced uniformity [126]. New synthesis methods that can avoid NGs’ scalability and introduce industrial-level amounts are current challenges [131,132,133]. This inexpensive carbon nanomaterial can exist with a positive and negative charge, which is mainly used to remove heavy metals [134]. Graphene-based materials include pristine graphene, graphene oxide, reduced graphene oxide, graphene quantum dots, graphene nanoribbons, graphene nanoplatelets, and three-dimensional graphene foam. NGs can offer high electrical conductivity and surface-to-volume ratio resulting in significant altitude of the drug/compound-loading capacity and high mammalian cell internalization, particularly by the nanographene oxide particles (Figure 5) that are up-taken into the cell cytoplasm [135,136]. They are also known for their aqueous, colloidal stability [137], and the delivery of insoluble drugs/compounds [129,130,138]. Challenges such as NGs agglomeration in solution, due to van der Waals interactions, might impact the loaded drug/compound pharmacological behavior [133] and limit cell specificity (in case of chemotherapy or radiotherapy and cancer cells); in addition, unexplored toxicity issues must be of further research concerns [137,139].

In MAFLD, NGs’ synthesis was very limited to developing sensors as essential indicators for clinical diagnosis, and prognosis judgment, including environment-friendly immunosensor of leptin using porous graphene-functionalized black phosphorus and gold nanoparticles successfully [140]. The sensitive and selective biosensor was also reported for the fibroblast growth factor 21 as an important MAFLD biomarker [141], while there was no record on anti-MAFLD NGs as drug delivery systems or the synergetic impact of NGs alone on one or more MAFLD manifestation. 

### 4.2. Lipid-Based Formulations

#### 4.2.1. Liposomes

**Figure 6:**
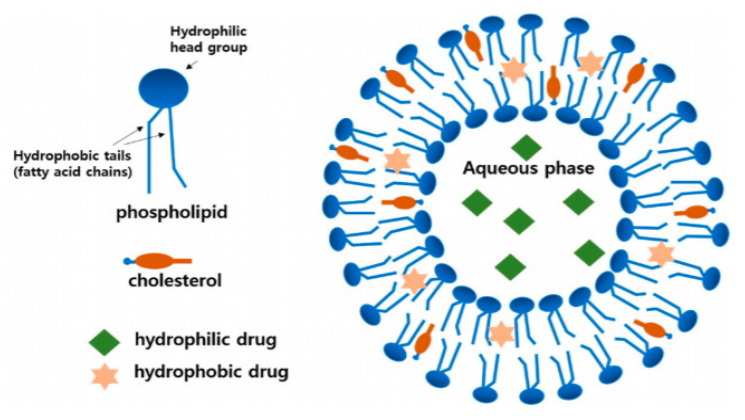
Structure of conventional liposome encapsulating hydrophilic and hydrophobic drugs. Adapted from the reference [152].

This nanocarrier is one of the first nanoformulations described in the early 1960s, with PS range of 0.025 μm to 2.5 μm [143,144]. Liposomes are non-toxic, flexible, biocompatible, completely biodegradable, and non-immunogenic nanocarriers that are suitable for systemic and non-systemic administrations. They offer significant advantages for hydrophilic and hydrophobic drug/compound delivery, including increment in efficacy stability, reduced toxicity, side effects, and targeting potentials. However, they are generally offering a short half-life due to oxidation and hydrolysis (particularly the negatively charged liposomes [145]), with possible drug/compound leakage, and high cost of production [146]. Parameters such as liposomes PS and bilayers’ number are critical as they control the circulation half-life and drug encapsulation percentage, respectively [144]. Thus, considerations are mainly given to its lipid composition, bilayer components, surface charge, size, and preparation method, which will significantly contribute to producing liposomes that can meet the set selection criteria [144]. The structural evolution of liposomes is illustrated in Figure 6.

Liposomes are prepared by passive or active loading techniques that include various mechanical dispersion methods [146,147]. In liver diseases, liposomes are promising nanocarriers for liver targeting as anticancer agent carriers [148,149]; this could be due to the fact that the liver exhibits the largest capacity for liposomal uptake, followed by the spleen among the other organs in the reticuloendothelial system [150]. In MAFLD therapeutics, liposome characteristics were mainly employed to enhance anti-MAFLD phyto- and conventional medicine properties. For instance, fenofibrate, an anti-dyslipidemia drug with prophylactic and/or inhibitory activity against inflammation, oxidation, and apoptosis, was loaded in liposomes. Fenofibrate is bio-pharmaceutically classified as a Class II drug, which means that, despite its high permeability, the drug’s poor solubility restricts its clinical outcomes [151]. Fenofibrate liposomes were prepared using a dry-film dispersing method with PS, Đ, and ZP of 122.1 nm, 0.293, and −2.92 mV, respectively. The EE% and drug-loading percentage were 96.6% and 7.44%, respectively, while the transmission electron microscope (TEM) images confirmed the spherical shape of the developed liposomes and the reported PS analysis by dynamic light scattering. The resulting liposomes offered a sustained in vitro release profile of fenofibrate, with a raised peak plasma concentration of 34.9-fold in comparison to the pure fenofibrate. Pharmacologically, fenofibrate liposomes (20mg/kg/day) reduced hepatic lipid accumulation significantly almost to the level of the control group of male C57BL/6 wild-type mice upon oral administration. Interestingly, unlike the pure fenofibrate, fenofibrate liposomes were able to remarkably reduce hepatic triglyceride content by 62.4% due to the increased oral absorption, which might offer a prophylactic effect against MAFLD.

Likewise, the flavonoid naringenin, which owns suggested anti-MAFLD activity due to its potent anti-inflammatory properties, was also successfully loaded (25 mg) in liposome by thin-film rehydration method, producing a liposome with PS 98 nm, EE%, and drug-loading percentage of 96.66% and 8.43%, respectively, with most of the naringenin encapsulated in the formulation’s lipid bilayer. The sustained release of the naringenin-loaded liposomes was significantly higher (81.74%) than the crude naringenin (24.35%), and the pharmacokinetic results on male C57BL/6J mice reveal the increase in maximum serum concentration (Cmax) by 6.7-fold. This naringenin liposome at 25 mg reduced the ALT, AST levels, and liver lipid accumulation in MAFLD induced by methionine choline-deficient diet compared to 100 mg of crude naringenin for the same reduction impact [153]. 

#### 4.2.2. Hybrid Lipid–Polymer Nanoparticles

**Figure 7:**
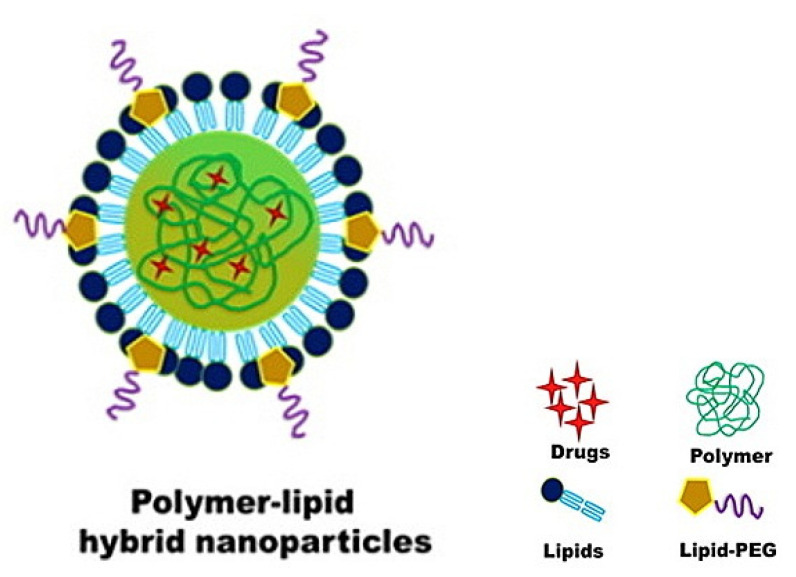
The structure of hybrid lipid–polymer nanoparticles. Adapted from the reference [170].

Hybridization is defined as a material involving two or more types of chemical bonds formed by “hybridization” of two or more monolithic materials [154]. The principle of hybrid-based NPs is emerging with some advanced nanosystems that were first reported in 2012 and 2014 [155,156,157]. The hybrid lipid–polymer nanoparticles (HLPNPs) are a novel generation core-shell nanostructure, notionally derived from both polymeric NPs and liposomes, where a polymer core remains enveloped by a lipid layer [158], as illustrated in Figure 7. This two-in-one nanosystem exists in both liquid or solid status, offering a drug control release profile [159], and owning a spherical shape and an outer surface that can be decorated in multifarious ways for active targeting of different approaches, including the smart delivery of DNA and RNA [158]. HLPNPs have two main methods of preparation, the Two-Step method, which usually yields PS between 200 nm and 400 nm and involves the preparation of polymeric NPs and lipid vesicles separately [158], and the One-Step method, where the separate preparation of the polymeric NPs and lipid vesicles is not a prerequisite. Other methods of preparation are still evolving, such as the use of bath sonication approach with the One-Step method (PS < 100 nm) [160], or the micro-vortex platform (PS ~30 nm to 170 nm) [161], in addition to the nanoprecipitation technique (PS ~60 to 190 nm) [134]. Thus, the selection of the right technique influences various parameters, such as size, dispersity, and shape [162,163,164]. The involved polymers in this NP synthesis are either previously reported in other nanosystems including Polystyrene, Maltodextrin, PLGA, Poly(ethylene glycol) monomethyl ether-Polylactic acid (mPEG-PLA), or Polylactic acid (PLA) alone [165], or a specifically functionalized polymer such as PFBT (poly[(9,9-dioctylfluorenyl-2,7-diyl)-alt-co-(1,4-benzo-(2,1′,3)-thiadiazole)]) [166], and poly-beta amino esters (PBAEs) [167]. Factors such as the core-shell nature, the polymer–oil ratio and the compatibility between encapsulated drug and dispersed oil are critically influencing the hydrodynamic characteristics and drug entrapment capacity of the hybrid matrix [157]. HLPNP accumulation in the liver is still controversial, for instance, solutol-HLPNPs showed low drug accumulation, in contrast, vitamin E or D-ɑ-tocopheryl polyethylene glycol succinate (TPGS) HLPNPs demonstrated high drug accumulation, which may be attributed to variations in solubility between the loaded materials and/or the existence of an inverse correlation between increased blood retention of HLPNPs and their accumulation in the liver [168]. 

In MAFLD nanomedicine discovery, recent research studied the value of developing silymarin-HLPNPs for better hepatoprotective efficacies upon enhancing silymarin low oral bioavailability (0.73%), poor aqueous solubility, and low membrane permeability. Silymarin was loaded into a previously developed HLPNP with PLGA alone [169], and compared with the modified silymarin-HLPNPs with PLGA and chitosan using a modified nanoprecipitation technique. It was noticed that the loaded HLPNPs with PLGA increased PS and Đ from 125.8 nm to 286.5 nm, and 0.142 to 0.226, respectively, upon hybridization with chitosan, while the ZP charge changed from negative (−43.1 mV) to positive (45.3 mV) due to chitosan presence. The X-ray diffraction confirmed silymarin dispersion within the developed nanoformulations. As for the EE%, there were no significant changes upon hybridization (from 97.39 to 97.05%).

The hybridization with chitosan had enhanced silymarin cellular uptake by human epithelial colorectal adenocarcinoma cells (Caco-2) and HepG2 cells and boosted its lipid-lowering effect and the triglyceride content in HepG2 cells in comparison to PLGA hybridization alone. Both formulations offered a burst release drug delivery, and the relative bioavailability study on healthy male Wistar rats with an oral dose of 20 mg/kg showed that silymarin-HLPNP with PLGA and chitosan offered a bioavailability of 1.23-fold and 14.38-fold higher than that of silymarin-HLPNP with PLGA alone, and pure silymarin suspension, respectively. The hepatoprotective and antihyperlipidemic effects of silymarin-HLPNP with PLGA and chitosan in MAFLD conditions were suggested through reducing the AST and ALT serum levels significantly in PNPLA3 I148M transgenic male and female mice with notably less macrovesicular steatosis compared to the group treated with pure silymarin suspension [97].

#### 4.2.3. Solid Lipid Nanocarriers

**Figure 8:**
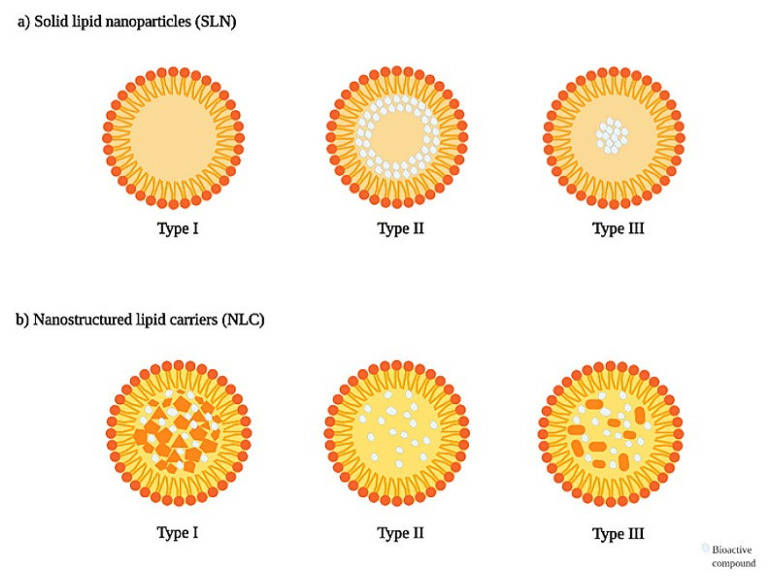
Classifications of (**a**) solid lipid nanoparticles (SLNs); (**b**) nanostructured lipid carriers (NLCs). Adapted from reference [172].

Solid lipid nanocarriers (SLNs) are colloidal drug delivery systems that were developed in the late 1980s. They are best described as a combination of liposomes and niosomes containing phospholipids and surfactant molecules, with a PS range from 40 to 1000 nm [171,172]; they are derived from oil-in-water (O/W) emulsions by replacing liquid lipids with a lipid matrix that is solid at room and body temperatures [173]. The lipid core typically consists of fatty acids (e.g., stearic acid), monoglycerides (e.g., glycerol monostearate), diglycerides (e.g., glycerol behenate), triglycerides (e.g., tripalmitin, tristearin, trilaurin), waxes (e.g., cetyl palmitate), or steroids (e.g., cholesterol), and is stabilized by appropriate surfactants [174,175]. Similar to most of the lipid-based formulations, SLN successfully delivered phytomedicines, proteins, and peptides, as well as a wide variety of genes and drugs. SLNs are classified into three types I, II, and III. As illustrated in Figure 8, type I is a homogeneous matrix model where the drug is dispersed in the lipid core with controlled release properties, while in type II a drug-free lipid core is formed, and a solid exterior shell with both lipid and drug is formed. Unlike type II, type III is adequate in achieving a prolonged drug release [172]. 

SLN has a wide range of advantages over the other nanosystems, precisely polymeric NPs and liposomes in terms of safety and owning the options of excluding organic solvents from excipients when desired, respectively. In addition, SLN offers excellent reproducibility and feasible large-scale production upon using the cost-effective high-pressure homogenization method, with a relative enhancement in loaded materials’ physicochemical stability compared to their pure form, along with biodegradable and cytocompatible natures. This nanosystem offers significant loading capacity with site-specific targeting capabilities and a controlled release profile [176]. An initial study suggests that SLN parenteral administration can bypass the gastro-intestinal route if the drug is pH sensitive, which may eventually result in high concentrations of drugs in the liver [177], but further investigations on SLN allocation and its benefit to liver and MAFLD is recommended. 

Main critical challenges should be taken into consideration when choosing the SLN preparation process, as each method produces SLNs with different morphological characteristics [178], and has its own challenges, including the possibility of drug expulsion from nano-vehicles and unsuitability to encapsulate hydrophilic materials, machine(s) unavailability in each laboratory, instability due to relatively high dispersion and metal contamination, the use of organic solvents, and limitation related to material solubility in carbon dioxide upon using hot homogenization technique, cold homogenization technique, ultrasonication or high-speed homogenization, solvent emulsification/evaporation method, and supercritical fluid extraction of emulsion method, respectively [179,180]. 

In MAFLD therapy, SLN was reported in the enhancement of berberine solubility (very slightly soluble in water) [181], which is a major component of *Coptis chinensis*. It is believed to have potential anti-MAFLD activities through multi mechanisms, including mediating insulin resistance, regulating the AMP-activated protein kinase pathway, and modifying the gut microenvironment. SLNs were prepared using the authors’ previously patented method that yielded SLNs with PS, Đ, and ZP of 76.8 nm, 0.402, and 7.87 mV, respectively. However, under TEM, while the particles were reported to have spherical shapes, with PS ranging from 50 to 150 nm, the XRD and NMR measurements ensured that berberine was dispersed and wrapped in the lipid carrier. Pharmacokinetically, and upon administrating berberine in pure and loaded SLNs forms at 50 mg/kg to different groups of male Sprague Dawley male rats, Cmax was increased by 3 folds, indicating that SLNs could minimize fluctuations in drug concentrations, promote absorption, and possess a slow-release character [182]. Berberine-SLNs at 100 mg/kg dosage showed more potent hypoglycemic effects than the equivalent dose of berberine alone in db/db male mice, especially in improving glucose tolerance and insulin sensitivity. In addition, it has uniquely suppressed the gain of body weight and offered hepatoprotective effects by lowering the ALT serum level and hepatic triglyceride content [183]. The authors urged for preclinical studies of berberine-SLNs with further chemical and physical characterization as well as safety assessments to ensure the availability of the berberine-SLNs in the market.

#### 4.2.4. Self-Emulsifying Drug Delivery System and Nano-Structured Lipid Carrier

These two lipid-based formulations’ application impact on MAFLD therapy was also limited to enhancing drug/compound physicochemical properties. This is due to their superior abilities in overcoming the leading factors behind insufficient oral bioavailability of lipophilic materials, including drugs efflux through P-glycoprotein and their first pass elimination due to metabolism through cytochrome P450 [184,185].

**Figure 9:**
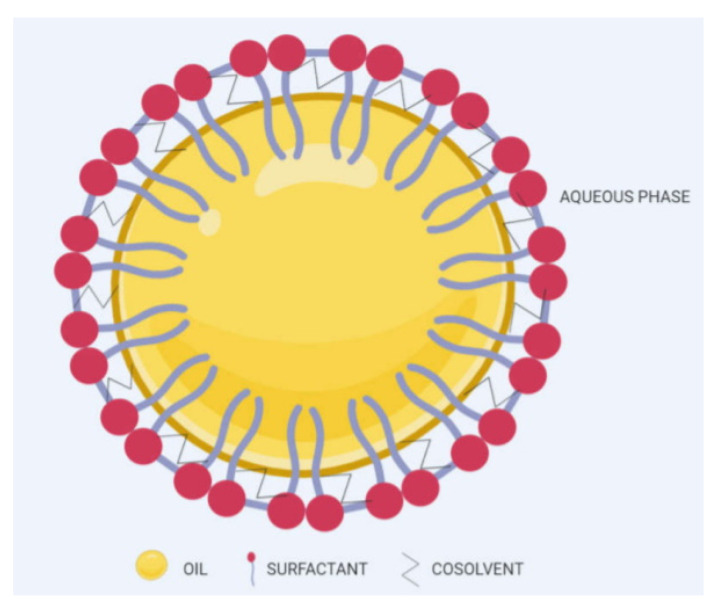
The typical self-emulsifying drug delivery system (SEDD) structure after dispersion in aqueous phase. Adapted from reference [200].

Self-emulsifying drug delivery systems (SEDDs) (Figure 9) have sparked profound interest from pharmaceutical researchers and industries through mainly antibiotic and antiviral SEDDs products in the market [186]; this micro/nanosystem offers droplet sizes of 5 µm to 200 nm [187] and <100 nm as well [188]. It can enhance the oral delivery of various therapeutic agents with different physicochemical properties [189,190], with either initial in vitro burst release, followed by a gradual-release phase or a sustained release profile [191,192,193,194]. SEDDs exist as liquid and solid formulations, where the solid form is suggested to provide better stability, reproducibility, and patient compliance, in addition to ease of process control, and various pharmaceutical dosage forms production (powders, capsules, tablets, or pellets) with the possibility of developing a controlled in vitro release attitude by mixing these dosage forms with suitable polymers or coating with polymeric films [193,195].

They are mainly composed of oil, surfactant, and cosurfactant phases mixed using the aqueous titration method, and the self-emulsification area is selected from the constructed pseudo-ternary diagram. The use of GRAS-type excipients with the least possible surfactant and cosurfactant (or co-solvent) ratios should be taken into consideration to assure that the final formulation is cytocompatible. As with other lipid-based formulations, the particle size relies on the excipients’ hydrophile–lipophile balance (HLB) value, where it is usually found that the higher the HLB value is, the lower the PS and Đ are, while the ZP charge is usually negative due to the presence of anionic surfactants and fatty acids unless specific lipids that grant the positive charge to the developed system are specifically employed, such as cationic surfactants, namely, tertiary amine surfactant, oleylamine, and the quaternary ammonium surfactants or polymers (e.g., Eudragit RS or RL) [196,197]. Incipient reports suggested that SEDDs could increase materials’ liver uptake [198,199], with further studies recommended on the cytocompatibility and mechanism behind such increment.

On the same line, nanostructured lipid carrier (NLC) is the enhanced version of SLN, as it outweighs the drawbacks of SLN. This includes loading capability issues by conceiving a less organized solid lipid matrix via blending a fluid lipid with the solid lipid, which provides less drug expulsion during storage [201,202,203]. NLC’s usual diameter ranges from 10 to 1000 nm, but most sizes between 50 and 300 nm are recommended for an easier crossing of barriers, increased uptake in cells and rapid action. Whereas NLCs usually own a controlled release profile, NLCs with size above 300 nm suggested providing a sustained drug delivery [204,205]. The particle size of this formulation is affected by several factors, including compositions’ properties, manufacturing process, processing temperature, pressure and number of cycles during high-pressure homogenization, sterilization and lyophilization [204]. They are mainly prepared by three different methods: the high-energy (homogenizer, sonicator, or microwave), the low-energy (microemulsion and double emulsion), and the organic solvent-based preparation [206]. They are primarily composed of lipids (solids and liquids at room temperature), surfactants, and co-surfactants, and other ingredients such as organic salts and ionic polymers. NLC offers significant chemical stability of the incorporated materials and effortless production possibilities on a large scale granting more affordable nanosystems than the polymeric NPs. The NLC classes are based on the structure of the formed crystals based on the lipid content, as explained in Figure 7. NLC type I offers an imperfect crystal model due to the presence of sufficient mixture amounts of liquid lipids and solid lipids, where the drug can place itself; NLC type II, known for its amorphous model, is linked to the use of special lipids that do not recrystallize after homogenization and cooling, which in turn will minimize drug expulsion. NLC type III, or the multiple models, shows a phase separation due to its composition of small oil nanoparticles that are inside the solid lipid matrix; this type is usually produced upon mixing solid lipids with higher amounts of oils in a ratio where the solubility of the oil in the solid lipid is exceeded [172]. In addition, NLC is also able to load lipophilic and hydrophilic materials, with no need to use organic solvents, offering control, and targeted drug release profile; however, as with most of the lipid-based formulations, inappropriate lipid/surfactant selection can lead to issues of stability and cytocompatibility [207]. As reported in SLN, NLC parenteral administration is recommended in liver delivery [177], as it demonstrated excellent liver tumor targetability on the in vivo level [208,209].

In the MAFLD drug discovery, silibinin’s oral bioavailability (flavonolignans) was enhanced with better efficacy to treat obesity-induced MAFLD upon loading it in different lipid-based nanocarriers, including SEDDs and NLCs [210]. The characterizations of SEDDs and NLCs showed that they had PS, Đ, and ZP of 83.89 nm and 25.49 nm, 0.31 and 0.23, and −8.55 mV and −34.27 mV, respectively. However, upon examining these formulations under TEM, the PSs were spherical but larger than the values by the Zetasizer (150 and 300 nm, respectively). The authors suggested that this is due to the additional preparations that the samples went under for TEM screening. Furthermore, silibinin’s loading capacity inside SEDDs and NLCs was significantly high, with 89.21% and 93.55%, respectively. Interestingly, SEDDs showed greater intensity and higher uptake in GIT than silibinin aqueous suspension and NLCs, probably due to SEDDs prolonged residence time in GIT. On the in vivo level, these lipid nanocarriers oral administration to male Sprague Dawley rats at a dose of 0.32 mg/kg could reduce the pathological signs of liver steatosis when compared to Roux-en-Y gastric bypass surgical option and upon treating MAFLD caused by obesity.

Recent successful application of NLC in MAFLD therapeutics was also reported using naringenin, a natural dihydroflavone that widely exists in citrus plants, with previously reported intense anti-inflammatory activity. Naringenin at 100 mg/kg/day attenuates MAFLD in male C57BL/6 wild-type mice via down-regulating the NLRP3/NF-κB signalling pathway [211]. The naringenin-loaded NLC was prepared by emulsion evaporation and solidification techniques to overcome its crude form poor water solubility (0.072 mg/mL), susceptibility to oxidation, and low oral bioavailability [212]. Upon characterization, the PS, ZP, EE%, and drug loading were 162.9 nm, −6.4 mV, 94.5%, and 22.5%, respectively, with good stability at 4 °C for 7 days. Naringenin-NLC cellular permeability was significantly higher than the naringenin alone on the Madin-Darby Canine Kidney (MDCK) cell line model, suggesting that naringenin could be a substrate of P-glycoprotein. The same enhancement profile was reported on the absorption in either ileum or jejunum of SD bio-breeding male rats. This could be why crude naringenin and naringenin-NLC had the same pharmacokinetic profile but at 100 mg/kg/day and 12.5 mg/kg/day, respectively. Interestingly, in MAFLD-induced mice, only a significant reduction in triglyceride levels was reported upon the oral intake of naringenin-NLC (12.5 mg/kg/day) in comparison to naringenin (100 mg/kg/day). Such a reduction pattern was not seen in ALT and AST levels, despite the fact that naringenin-NLC (12.5 mg/kg/day) recorded the highest quantification concentrations in the liver compared to crude naringenin at 50 and 100 mg/kg/day.

#### 4.2.5. Nanoemulsion

**Figure 10:**
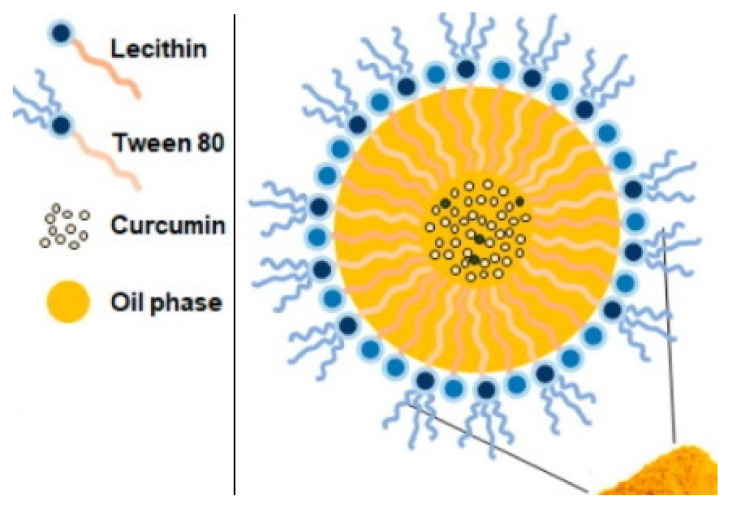
The structure of nanoemulsion droplet, consisting of lecithin as an emulsifier dissolved in oil phase, and Tween 80 as another emulsifier dissolved in aqueous phase, while the active material is curcumin. Adapted from reference [222].

Nanoemulsions (NEs) are characterized as colloidal systems with droplet sizes ranging from ~50–500 nm, low viscosity, transparency and spherical shape. They are designed to enhance a variety of compounds’ functional and physicochemical properties and shelf life, as well as cosmetical, nutritional, and pharmacological values. NEs can be delivered through the conventional and novel routes of administration, including transmucosal and transdermal routes [213,214]. This nanosystem offers a sustained release drug delivery [215,216], while the small droplet size promotes the material burst release pattern from NEs [217]. NEs’ release profile is directly proportional to its droplet size, smaller droplet size, and larger interfacial area, which promotes rapid drug release [218,219]. It consists of the main three phases, the oil, the aqueous phase and the emulsifier phase, as explained in Figure 10. The oil phase could be made of triacylglycerols, diacylgycerols, monoacylglycerols, and free fatty acids, non-polar essential oils, mineral oils, lipid substitutes, waxes, and oil-soluble vitamins.

Oil phase selection significantly influences the formulation’s stability and characteristics. The production of homogenous NEs relies on the preparation method using mainly either high or low energy methods (e.g., phase inversion temperature, phase inversion composition, ultrasonication, high-pressure homogenization and microfluidization, etc.); such selection is fundamentally reflected on the NEs’ loading, encapsulation efficiency, PS and Đ [213,220]. Usually, NEs are confused with microemulsions due to significant similarities between the two systems in terms of their physical appearance, components, and preparation techniques. However, NEs are kinetically stable and thermodynamically metastable, while microemulsions are thermodynamically stable [221]. 

NEs and NLC surpassed SLN in terms of bioaccessibility and small droplet size output (<100 nm), while NEs offer higher release rates for oral or topical routes [223,224], with equal abilities of NEs and NLC to provide protection and stability against UV radiation damages [225]. NEs were also found to be superior to SLN and polymeric nanosuspension when enhancing drug’s solubility and dissolution rate [226]. Despite the various advantages of NEs, the safety concerns associated primarily with the use of synthetic emulsifiers are a crucial problem to be tackled. Most of the synthetic emulsifiers can trigger toxic symptoms with the prolonged administration, including the potential binding of anionic emulsifiers to proteins, enzymes, and phospholipid membranes in the human body, resulting in various adverse alterations, such as dysfunction of enzymes, modification of protein structure, and phospholipid in the membrane cell [227]. Accordingly, replacing the synthetic emulsifiers with natural substitute(s) is one of the on-demand novelties in the NEs’ construction. Initial studies show that NEs’ intravenous administration provided drug delivery to various organs, including the liver, with promising targeting effects [228,229], while other studies indicate a preferential liver uptake to the oral NEs [230,231,232].

In MAFLD cases, the fat-soluble vitamin D (cholecalciferol) deficiency was observed. It is known for its anti-inflammatory, antioxidant and immune-modulating activity; however, the intake of conventional vitamin D is associated with variable oral bioavailability, poor water solubility and sensitivity to environmental factors, such as light, oxygen, and heat. The vitamin D NEs were prepared using the high-intensity ultrasonication method to overcome these physicochemical drawbacks through employing purposely designed pea protein natural amphiphilic surfactant resulting in droplet size of 88.90 nm, and EE% of 93.2% with a significant UV light stability for 180 min (remaining 74.22% of vitamin D in NEs) in comparison to the pure vitamin D (remaining 8.71%) [233]. The authors proposed that the light stability could be due to the presence of the aromatic side chains and double bonds in pea proteins which might absorb UV light. The digestion test for this oral NE showed good recovery of vitamin D after three hours (62.9%). As an anti-MAFLD, this NE demonstrated superiority to the conventional vitamin D in terms of reducing elevated liver enzymes, improved lipid profile, enhanced fatty acid oxidation and attenuates liver inflammation and fibrosis in high-fat diet male albino rats, suggesting a remarkable hepato-protectivity effect of the developed NEs [234]. 

#### 4.2.6. Micelles

**Figure 11:**
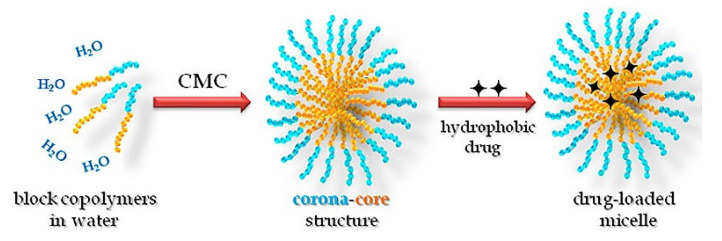
Schematic representation for the micellization of diblock copolymers and drug encapsulation in polymeric micelle. CMC: critical micelle concentration. Adopted from reference [245].

Micelles are self-assembling colloidal dispersions with a hydrophobic core and hydrophilic shell that have a PS ranging from 10 to 100 nm [235,236]. For the hydrophilic materials and proteins, the reverse micelles are the more suitable carriers [237,238], while the unimolecular micelles that are composed of block copolymers (i.e., core(laur)-polyethylene glycol) offer a much more thermodynamic stable micelle form [239], as explained in Figure 11. These polymers have several hydrophilic and hydrophobic regions in one molecule, which enables the self-assembly of one molecule into a micelle. Micelles are known for their high drug content and kinetic stability [240], and usually they are administrated through topical, and ophthalmic routes, but they could also be taken orally and intravenously. Micelles are mainly composed of the amphiphilic molecule (surfactants and copolymers), and they are favorable for their simple preparation procedure that offers increased drug solubility, reduced toxicity, increment in circulation time, enhanced tissue penetration and targetability [241], particularly upon using pH-sensitive polymers [242]. Care must be given when designing this nanocarrier in terms of assuring drug system stability, excipients-drug/compound compatibility, particularly for hydrophilic ones, and the suitability of the short-sustained release profile offered by micelles to the aim of the study [243]. The most controlling experimental factors upon designing micelles are the degree of swelling of the corona, excipient’s concentration, temperature, pH, ionic strength, and sample preparation. Polymeric micelles will have additional factors impacting the micelles characteristics, mainly PS, including the polymer/copolymer molecular weight, the relative proportion of hydrophilic and hydrophobic chains, and the quantity of solvent trapped inside the micellar core [244].

Micelles have surpassed other nanosystems for liver targeting therapy in terms of ideal distribution and relatively proper staying duration in the site upon employing smart excipients such as arabinogalactan polymer [246]. Polymeric micelles exhibited a high capacity to incorporate various bioactive molecules such as antisense oligonucleotides, plasmid DNA, proteins, small interfering ribonucleic acids (siRNAs), messenger RNAs [247]. Micelles could offer positive liver-targeting, mainly for anticancer agents [246,248,249], with further research needed in the anti-MAFLD future. 

On the nano-molecular level, loading GDC-0449 and miR-29b1 mimics in micelles significantly reduced the collagen deposition in the liver, which in turn improved the liver fibrosis cases, suggesting that these micelles represent a promising candidate in treating MAFLD. The GDC-0449 and miR-29b1 mimics are a hedgehog ligand inhibitor that plays a vital role in treating liver fibrosis by inhibiting several pro-fibrotic genes. The measurements of PS, Đ and ZP were 80 nm, 0.2 and −0.5 mV, respectively, while the TEM image showed micelles’ spherical shape and well-dispersed particles, with one-week stability upon placing the micelles on the bench, in addition to 24 h stability in the serum. Moreover, the cellular uptake study indicated that micelles could transfect miRNA efficiently in the immortalized rat liver stellate cell line (HSC-T6), even in the presence of serum, which indicates the in vivo applicability of these micelles over many of the commercially available transfection reagents [250].

#### 4.2.7. Nanocrystals

**Figure 12:**
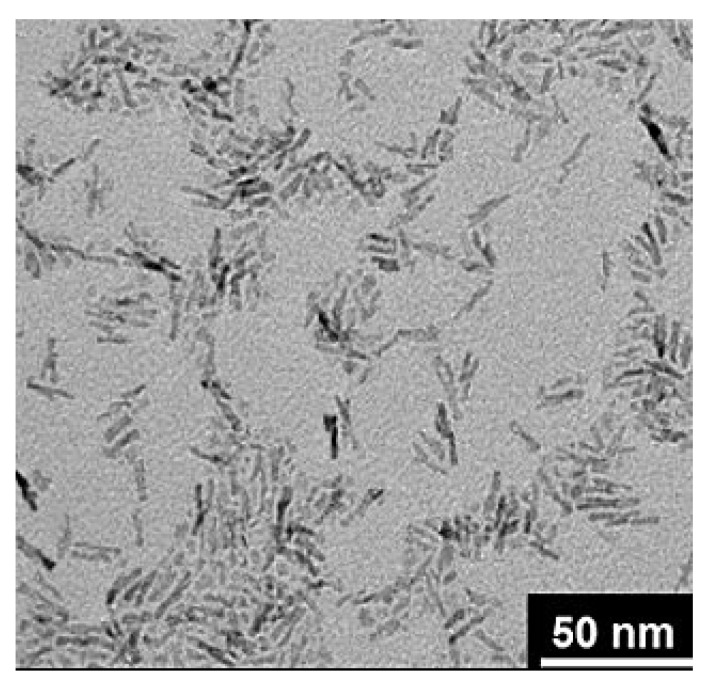
Transmission electron microscopy image of a typical nanocrystal sample loaded with titanium dioxide. Adapted from the reference [268].

Nanocrystals (NCs) are currently considered as a promising drug delivery system (PS < 1 μm) with more than twenty approved formulations in the market including those that can treat MAFLD accompanied manifestations of hypercholesterolemia, diabetes, and inflammation [251,252,253]. NCs’ structure has various shapes, including dot, sphere, cube, rod, triangle, and hexagon [254]; an example of it, expressed under TEM, is in Figure 12. The advantages of NCs are focused on overcoming erratic absorption of poorly soluble drugs by significantly improving drug/component solubilization and bioavailability ratios which have a positive effect on their pharmacokinetics and therapeutic applications, as well as surface/cell membrane adhesion [251,253,255]. This nanosystem excels above the previously reported nanocarriers in terms of drug-loading capacity, and delivery efficiency to cells or tissues, which in turn lead to much more desirable pharmacological activities as compared to nanocapsules [256], liposomes [257], SLN, NLC [258], and NEs [259]. NCs are primarily administered via oral, parenteral, pulmonary, ocular and topical routes, using different methods of preparations depending on the desired final NC specifications where the combination technique is used to produce NCs with PS < 100 nm [260]. Furthermore, the bottom-up method is for laboratory-scale use [261], high-pressure homogenization for PS > 100 nm [262] and media milling for less contamination and pharmaceutical industry scale production [263]. NCs with an amorphous crystalline substructure have an increased dissolution rate and are better suited for the delivery of highly hydrophobic rather than hydrophilic drugs, with the ability to protect drugs from environmental conditions and to provide a controlled release profile. Critical steps to study during NC formulation include the freeze-drying process and dependence on critical microcell concentrations for improved stability and drug-loading capacity [264]. At the cellular level, NC charge plays a crucial role in their efficiency, where positive NCs can further interact with negatively charged organelles and DNA, leading to higher cell uptake and cytotoxicity compared to negatively charged particles. However, negatively charged NCs also exhibit significant uptake through clathrin- and caveolae-mediated uptake mechanisms [265,266,267].

Although NCs were effective in improving the drug delivery and pharmacological outcomes of cardiovascular, ophthalmic, viral, helminthic, bacterial, inflammatory and cancer diseases [251], NCs impact on liver disease is not well explored; it is proposed that oral and injected NCs might offer liver targeted drug delivery options [269,270]. As for directly addressing MAFLD, the anti-obesity and first time reported anti-inflammatory nanocrystalline cerium dioxide (CeO_2_) were suggested as part of MAFLD therapy based on CeO_2_ antioxidant auto-regenerative ability that was proven on the in vivo level [271,272,273]. These effects are linked to the significant ability of the NCs to reduce lipid peroxidation in the liver tissue, and to reversibly switch between Ce^3+^ and Ce^4+^ presented on its surface, resulting in the formation of oxygen defects in the crystal lattice that act as “reactive sites” or “hot spots” for free radical scavenging. In addition, CeO_2_-NCs reported reducing steatosis, lobular inflammation and pro-inflammatory cytokines (IL-1β, IL-12Bp40) in rat serum. Interestingly, CeO_2_-NCs also restored the level of anti-inflammatory cytokines (IL-4, IL-10, TGF-β) to the control values. Regarding the CeO_2_-NC formulation, details were limited to its PS of 4.9 nm with a ZP of-20 mV, while the X-ray diffraction is single-phase and corresponds to the cubic cerium dioxide [273]. 

## 5. Future Perspectives

In liver drug delivery, nanoformulations including liposomes, polymeric nanoparticles, and polymeric micelles are the primarily successful nanocarriers mainly in targeting hepatocytes selectively, enhancing loaded materials safety, stability, and physicochemical properties, such as solubility, which is limiting their pharmacological activities based on the drug’s biopharmaceutical classification [274,275]. As can be seen from our previous illustrations, additional detailed answers are needed to address the issues of the nanomedicine impact on MAFLD therapy from a variety of different perspectives, such as the logic behind the selection of a particular nanosystem, excipients, their ratios and preparation method(s). Selecting the route of administration is an additional issue that is critically linked to the type of nanocarrier, the efficiency and the nature of the loaded drug/compound. First and foremost, researchers should consider some key factors while designing each formulation. For example, lipid nanoparticles are known to promote lymphatic transport and can bypass the liver and avoid hepatic first-pass effect [276], which may lead to a conflict between in vitro and in vivo outcomes when using such nanocarriers. Although many studies report boosted in vitro dissolution rate and in vivo bioavailability of different drugs/compounds upon loading in lipid-based nanoparticles, further investigation is needed into the mechanism behind which physiological enhancement in the MAFLD-patient liver has occurred, even though the drug/compounds may be bypassing the liver. Another critical question to be answered is nanomedicine’s ability to overcome the absence of a single anti-MAFLD therapy.

In addition, a variety of nanocarriers should be considered to examine their unreported effects on MAFLD for therapeutic and diagnostic purposes, such as nanospheres, nanocapsules, nanosuspensions, niosomes, dendrimers, nanogels, carbon nanotubes, polymerized nanographene oxide particles, nanodiamonds, quantum dots, exosomes, polymers and nanoparticle-mediated targeted drug delivery systems, using specific ligands [133,135,136,140,277]. Future formulators are advised to use the concept of quality-by-design (QbD) formulation, as conventional pharmaceutical development processes are based on quality through testing, and are out of use [278].

It is essential to characterize the developed formulations through a boarder range of assays to clarify excipient–excipient, drug–excipient compatibility and long-term physicochemical stability. This includes fourier-transform infrared spectroscopy, nuclear magnetic resonance spectroscopy, differential scanning calorimetry, X-ray diffraction, and scanning electron microscopy. The effect of the nanosystem charge (positive and negative) on the safe and efficient delivery of anti-MAFLD liver drugs, as well as the mechanism of metabolism, excretion rate, and route, should be extensively studied. Unfortunately, when examining nanoformulations at the in vivo level, gender is an overlooked factor; future research should involve both female and male animals to compare their responses to the formulation with a toxicity profile.

Furthermore, many phytochemicals were recognized for their anti-MAFLD therapeutics as illustrated in Table 1, and yet, when their nanosystems were developed (if any), they were never evaluated for the anti-MAFLD activities. Future studies can therefore focus on assessing such already developed nanosystems using the proper in vitro, ex vivo and in vivo MAFLD models. Silybin-nanosuspension and micelles that improved the low oral absorption and bioavailability of silybin are examples of such instances. [279,280], as well as Schisandra extract loaded in liposome-encapsulated with β-cyclodextrin [281]. Natural polyphenol compounds are a major current trend in the development of MAFLD therapy [282,283,284,285,286,287] and have a strong presence worldwide in many traditional diets, mainly Mediterranean diets [288], and common Chinese dietary herbs [289]. Studies are limited on such phytomedicinal compounds having an impact on MAFLD when loaded into nanosystems, mainly flavonoids and phenolic acids that account for about 60% and 30% of the polyphenols, respectively [290]. In terms of folk medicine, curcumin was one of the most investigated compounds for liver hepato-protectivity and MAFLD treatment potentials. However, these investigations still did not cover most of the famous nanosystems. For instance, curcumin-loaded polymerized graphene oxide nanocarrier was reported to improve its sustained release behavior and bioavailability with a cytocompatible profile [291], while curcumin liposomes were reported to improve the oxidative stress/antioxidant balance and alleviate inflammation in experimental acetaminophen-induced hepatotoxicity [292]. Studying the impact of such nanosystems on MAFLD might grant answers to many questions concerning its generic drugs (anti-obesity, hypoglycemic, lipid-lowering, cytoprotective and antioxidant agents), and phytomedicinal therapies.

The use of synergistic excipients in the treatment of MAFLD was restricted to mostly chitosan polymers, so more research should consider investigating and/or using other synergistic excipients such as oils, surfactants, co-surfactants, co-solvents. Ginger and garlic oils are promising to begin with as they have been successfully used in the construction of different nanoemulsions for their anti-obesity, anti-hyperlipid activity, along with their hepatoprotective ability to minimize serum total cholesterol, low-density lipoproteins, very low-density lipoproteins, triglycerides, and atherogenic indices. In addition, the resultant and improved serum high-density lipoprotein cholesterol levels suggest that animals are on the way to recovery from MAFLD [313]. However, the ability of such smart excipients to produce nanosystems meeting the required selection criteria is a priority too.

Another overlooked consideration in MAFLD nanomedicine therapy is the fate of nanocarriers and related safety issues following administration through various routes, with reports of inflammation-related DNA damage in the liver associated with exposure to nanoparticles [314], in addition to a persistent rise in body weight and elevated blood glucose levels due to manganese oxide nanoparticle subcutaneous injection at 100 μg/kg, along with a drop in low-density lipoprotein levels [315]. On the same line, and as the liver is suggested to be the major organ for nanoparticle deposition after their absorption, a single oral dose of silver nanoparticles induced acute liver inflammation in healthy male mice; this emphasizes an increased threat to the susceptible overweight population [316]. Therefore, the sequence of safe, effective and stable novel nanoformulation should not be neglected.

## 6. Concluding Remarks

NAFLD has been re-identified and renamed to a precise name, MAFLD. The disease is escalating all over the world, burdening the health’s of millions due to its multiple manifestations of intrahepatic lipids, alterations of energy metabolism, insulin resistance, and inflammatory processes that contribute to the lack of a specific and effective single treatment. On the other hand, the proper selection and design of the nanocarrier, and it’s in vitro, ex vivo and in vivo evaluation models could provide a clearer vision about the possible solutions to the available conventional anti-MAFLD drugs and traditional herbal remedies in order to protect them from the harsh physiological or environmental conditions. The relation between enhanced materials’ solubility, dissolution rate, bioavailability, and MAFLD nanomedicine discovery is in need of further investigation, mainly when using lipid-based nanocarriers, where the possibility of drug bypassing the liver is high. In this regard, targeted drug delivery might be more promising in MAFLD therapy as exploring such approach could offer a single anti-MAFLD therapy. The fate of nanoformulations inside the body, including their metabolism, accumulation, and excretion, is a crucial issue to consider when designing such formulations to ensure formulations’ safety and efficacy at the same time.

## Figures and Tables

**Table 1:** Suggested phytochemicals for future anti-MAFLD nanocarrier research in relation to it being active and/or excipient with synergetic effects.

Phytochemical Compound Name	Source	Reported Activity	Reference
α-linolenic acid	Flaxseed	Weight loss, reducing the risk of atherosclerosis, diabetes, metabolic syndrome and dyslipidemia	[293,294]
Flavonoids, steroids, terpenoids and phenolic acids	Cinnamon	Antioxidant, anti-hyperglycemic effect, improves lipid profiles	[294,295]
Flavonoids (catechins)	Green Tea	Antioxidant	[294,296]
Isoflavones	Soybeans	Protection and treatment of cardiovascular diseases, diabetes, decreasing hepatic lipid deposition, and increasing antioxidant capacity	[294,297]
Glycyrrhizin	Licorice root	Reduce liver inflammation, antioxidant, anti-diabetic, reduce ALT, AST, and hepato-protective	[294,298]
Xuezhikang, unsaturated fatty acids, and flavonoids	Red yeast rice	Lowering serum total glycerides, total cholesterol	[294,299,300,301]
Qianggan	A combination of 16 Chines herbs	Anti-fibrotic	[294,302]
Chlorophyll, tocopherols, and ubiquinone	Chlorella green algae	Prevention and treatment of several metabolic disorders (e.g., dyslipidemia, hyperglycemia, hypertension, obesity)	[294,303]
Danning Pian	A combination of several herbs	Similar to ursodeoxycholic acid in terms of antioxidant and cytoprotective efficacy	[294,304]
Yiqi Sanju	A combination of several herbs	Improved the MAFLD grade	[294,305]
Anthocyanins and a variety of phenolic acids, including caffeic, ferulic, sinapic, and salicylic acids	Bayberry	High antioxidant and anti-inflammatory capacity	[294,306,307]
Dietary phospholipids (dilinoleoylphosphatidylcholine)	Soybeans	Anti-inflammatory, anti-fibrotic, and lipid and regulating effects	[294,308]
Allicin, and flavonoids	Garlic	Anti-hepatic oxidative stress potentials	[294,309,310]
Dihydromyricetin	Ampelopsis grossedentata	Antioxidant, anti-inflammatory, hepatoprotective and glucose-regulatory activities	[294,311]
Phyllanthus	Phyllanthus urinaria	Hepatoprotective and strong antioxidant capacities	[294,312]

## References

[1] Basaranoglu M., Neuschwander-Tetri B.A. (2006). Nonalcoholic Fatty Liver Disease: Clinical Features and Pathogenesis. Gastroenterol. Hepatol..

[2] Ray K. (2013). NAFLD—The next global epidemic. Nat. Rev. Gastroenterol. Hepatol..

[3] Younossi Z., Anstee Q.M., Marietti M., Hardy T., Henry L., Eslam M., George J., Bugianesi E. (2018). Global burden of NAFLD and NASH: Trends, predictions, risk factors and prevention. Nat. Rev. Gastroenterol. Hepatol..

[4] Sarin S.K., Kumar M., Eslam M., George J., Al Mahtab M., Akbar S.M.F., Jia J., Tian Q., Aggarwal R., Muljono D.H. (2020). Liver diseases in the Asia-Pacific region: A Lancet Gastroenterology & hepatology Commission. Lancet Gastroenterol. Hepatol..

[5] Fouad Y., Waked I., Bollipo S., Gomaa A., Ajlouni Y., Attia D. (2020). What’s in a name? Renaming ‘NAFLD’ to ‘MAFLD’. Liver Int..

[6] Åberg F., Helenius-Hietala J., Puukka P., Färkkilä M., Jula A. (2018). Interaction between alcohol consumption and metabolic syndrome in predicting severe liver disease in the general population. Hepatology.

[7] The Lancet Gastroenterology Hepatology (2020). Redefining non-alcoholic fatty liver disease: What’s in a name?. Lancet Gastroenterol. Hepatol..

[8] Eslam M., Sanyal A.J., George J., Sanyal A., Neuschwander-Tetri B., Tiribelli C., Kleiner D.E., Brunt E., Bugianesi E., Yki-Järvinen H. (2020). MAFLD: A Consensus-Driven Proposed Nomenclature for Metabolic Associated Fatty Liver Disease. Gastroenterology.

[9] Tilg H., Effenberger M. (2020). From NAFLD to MAFLD: When pathophysiology succeeds. Nat. Rev. Gastroenterol. Hepatol..

[10] Younossi Z.M., Blissett D., Blissett R., Henry L., Stepanova M., Younossi Y., Racila A., Hunt S., Beckerman R. (2016). The economic and clinical burden of nonalcoholic fatty liver disease in the United States and Europe. Hepatology.

[11] Ludwig J., Viggiano T.R., McGill D.B., Oh B.J. (1980). Nonalcoholic steatohepatitis: Mayo Clinic experiences with a hitherto unnamed disease. Mayo Clin. Proc..

[12] Lee R.G. (1989). Nonalcoholic steatohepatitis: A study of 49 patients. Hum. Pathol..

[13] Laurin J., Lindor K.D., Crippin J.S., Gossard A., Gores G.J., Ludwig J., Rakela J., McGill D.B. (1996). Ursodeoxycholic acid or clofibrate in the treatment of non-alcohol-induced steatohepatitis: A pilot study. Hepatology.

[14] Arshad T., Golabi P., Paik J., Mishra A., Younossi Z.M. (2018). Prevalence of Nonalcoholic Fatty Liver Disease in the Female Population. Hepatol. Commun..

[15] Wang Z., Xu M., Hu Z., Shrestha U.K. (2015). Prevalence of nonalcoholic fatty liver disease and its metabolic risk factors in women of different ages and body mass index. Menopause.

[16] Williams C.D., Stengel J., Asike M.I., Torres D.M., Shaw J., Contreras M., Landt C.L., Harrison S.A. (2011). Prevalence of nonalcoholic fatty liver disease and nonalcoholic steatohepatitis among a largely middle-aged population utilizing ultrasound and liver biopsy: A prospective study. Gastroenterology.

[17] Browning J.D., Szczepaniak L.S., Dobbins R., Nuremberg P., Horton J.D., Cohen J.C., Grundy S.M., Hobbs H.H. (2004). Prevalence of hepatic steatosis in an urban population in the United States: Impact of ethnicity. Hepatology.

[18] Bacon B.R., Farahvash M.J., Janney C.G., Neuschwander-Tetri B.A. (1994). Nonalcoholic steatohepatitis: An expanded clinical entity. Gastroenterology.

[19] Ballestri S., Nascimbeni F., Baldelli E., Marrazzo A., Romagnoli D., Lonardo A. (2017). NAFLD as a Sexual Dimorphic Disease: Role of Gender and Reproductive Status in the Development and Progression of Nonalcoholic Fatty Liver Disease and Inherent Cardiovascular Risk. Adv. Ther..

[20] Niaz A., Ali Z., Nayyar S., Fatima N. (2011). Prevalence of NAFLD in Healthy and Young Male Individuals. ISRN Gastroenterol..

[21] Mencin A.A., Lavine J.E. (2011). Advances in pediatric nonalcoholic fatty liver disease. Pediatr. Clin..

[22] Vajro P., Lenta S., Socha P., Dhawan A., McKiernan P., Baumann U., Durmaz O., Lacaille F., McLin V., Nobili V. (2012). Diagnosis of nonalcoholic fatty liver disease in children and adolescents: Position paper of the ESPGHAN Hepatology Committee. J. Pediatr. Gastroenterol. Nutr..

[23] Nobili V., Alisi A., Valenti L., Miele L., Feldstein A.E., Alkhouri N. (2019). NAFLD in children: New genes, new diagnostic modalities and new drugs. Nat. Rev. Gastroenterol. Hepatol..

[24] Paik J.M., Henry L., De Avila L., Younossi E., Racila A., Younossi Z.M. (2019). Mortality Related to Nonalcoholic Fatty Liver Disease Is Increasing in the United States. Hepatol. Commun..

[25] Estes C., Chan H.L.Y., Chien R.N., Chuang W.-L., Fung J., Goh G.B.-B., Hu T.H., Huang J.-F., Jang B.K., Jun D.W. (2020). Modelling NAFLD disease burden in four Asian regions-2019-2030. Aliment. Pharmacol. Ther..

[26] Estes C., Anstee Q.M., Arias-Loste M.T., Bantel H., Bellentani S., Caballeria J., Colombo M., Craxi A., Crespo J., Day C.P. (2018). Modeling NAFLD disease burden in China, France, Germany, Italy, Japan, Spain, United Kingdom, and United States for the period 2016–2030. J. Hepatol..

[27] Marchesini G., Brizi M., Morselli-Labate A.M., Bianchi G., Bugianesi E., McCullough A.J., Forlani G., Melchionda N. (1999). Association of nonalcoholic fatty liver disease with insulin resistance. Am. J. Med..

[28] Buzzetti E., Pinzani M., Tsochatzis E.A. (2016). The multiple-hit pathogenesis of non-alcoholic fatty liver disease (NAFLD). Metabolism.

[29] El-Agroudy N.N., Kurzbach A., Rodionov R.N., O’Sullivan J., Roden M., Birkenfeld A.L., Pesta D.H. (2019). Are Lifestyle Therapies Effective for NAFLD Treatment?. Trends Endocrinol. Metab..

[30] European Association for the Study of the Liver (EASL), European Association for the Study of Diabetes (EASD), European Association for the Study of Obesity (EASO) (2016). EASL-EASD-EASO Clinical Practice Guidelines for the management of non-alcoholic fatty liver disease. J. Hepatol..

[31] Kwak M.-S., Kim D. (2018). Non-alcoholic fatty liver disease and lifestyle modifications, focusing on physical activity. Korean J. Intern. Med..

[32] Promrat K., Kleiner D.E., Niemeier H.M., Jackvony E., Kearns M., Wands J.R., Fava J.L., Wing R.R. (2010). Randomized controlled trial testing the effects of weight loss on nonalcoholic steatohepatitis. Hepatology.

[33] Dixon J.B., Bhathal P.S., Hughes N.R., O’Brien P.E. (2004). Nonalcoholic fatty liver disease: Improvement in liver histological analysis with weight loss. Hepatology.

[34] Yoo E.R., Sallam S., Perumpail B.J., Iqbal U., Shah N.D., Kwong W., Cholankeril G., Kim D., Ahmed A. (2018). When to Initiate Weight Loss Medications in the NAFLD Population. Diseases.

[35] Nakajima K. (2012). Multidisciplinary Pharmacotherapeutic Options for Nonalcoholic Fatty Liver Disease. Int. J. Hepatol..

[36] Garvey W.T., Mechanick J.I., Brett E.M., Garber A.J., Hurley D.L., Jastreboff A.M., Nadolsky K., Pessah-Pollack R., Plodkowski R. (2016). American Association of Clinical Endocrinologists and American College of Endocrinology Comprehensive Clinical Practice Guidelines for Medical Care Of Patients with Obesity. Endocr. Pract..

[37] Snyder H.S., Sakaan S.A., March K.L., Siddique O., Cholankeril R., Cummings C.D., Gadiparthi C., Satapathy S.K., Ahmed A., Cholankeril G. (2018). Non-alcoholic Fatty Liver Disease: A Review of Anti-diabetic Pharmacologic Therapies. J. Clin. Transl. Hepatol..

[38] Jalali M., Rahimlou M., Mahmoodi M., Moosavian S.P., Symonds M.E., Jalali R., Zare M., Imanieh M.H., Stasi C. (2020). The effects of metformin administration on liver enzymes and body composition in non-diabetic patients with non-alcoholic fatty liver disease and/or non-alcoholic steatohepatitis: An up-to date systematic review and meta-analysis of randomized controlled trials. Pharmacol. Res..

[39] Li Y., Liu L., Wang B., Wang J., Chen D. (2013). Metformin in non-alcoholic fatty liver disease: A systematic review and meta-analysis. Biomed. Rep..

[40] Nair S., Diehl A.M., Wiseman M., Farr G.H., Perrillo R.P. (2004). Metformin in the treatment of non-alcoholic steatohepatitis: A pilot open label trial. Aliment. Pharmacol. Ther..

[41] Haukeland J.W., Konopski Z., Eggesbø H.B., von Volkmann H.L., Raschpichler G., Bjøro K., Haaland T., Løberg E.M., Birkeland K. (2009). Metformin in patients with non-alcoholic fatty liver disease: A randomized, controlled trial. Scand. J. Gastroenterol..

[42] Blazina I., Selph S. (2019). Diabetes drugs for nonalcoholic fatty liver disease: A systematic review. Syst. Rev..

[43] Gastaldelli A., Harrison S.A., Belfort-Aguilar R., Hardies L.J., Balas B., Schenker S., Cusi K. (2009). Importance of changes in adipose tissue insulin resistance to histological response during thiazolidinedione treatment of patients with nonalcoholic steatohepatitis. Hepatology.

[44] Lutchman G., Modi A., Kleiner D.E., Promrat K., Heller T., Ghany M., Borg B., Loomba R., Liang T.J., Premkumar A. (2007). The effects of discontinuing pioglitazone in patients with nonalcoholic steatohepatitis. Hepatology.

[45] Tacelli M., Celsa C., Magro B., Giannetti A., Pennisi G., Spatola F., Petta S. (2018). Antidiabetic Drugs in NAFLD: The Accomplishment of Two Goals at Once?. Pharmaceuticals.

[46] Ganguli S., DeLeeuw P., Satapathy S.K. (2019). A Review of Current and Upcoming Treatment Modalities in Non-Alcoholic Fatty Liver Disease and Non-Alcoholic Steatohepatitis. Hepat. Med..

[47] Perelas A., Tsoulkani A., Perrea D. (2010). Effects of lipid-lowering drugs on adiponectin. Curr. Vasc. Pharmacol..

[48] Nascimbeni F., Pellegrini E., Lugari S., Mondelli A., Bursi S., Onfiani G., Carubbi F., Lonardo A. (2019). Statins and nonalcoholic fatty liver disease in the era of precision medicine: More friends than foes. Atherosclerosis.

[49] Argo C.K., Loria P., Caldwell S.H., Lonardo A. (2008). Statins in liver disease: A molehill, an iceberg, or neither?. Hepatology.

[50] Pastori D., Polimeni L., Baratta F., Pani A., Del Ben M., Angelico F. (2015). The efficacy and safety of statins for the treatment of non-alcoholic fatty liver disease. Dig. Liver Dis..

[51] Casula M., Mozzanica F., Scotti L., Tragni E., Pirillo A., Corrao G., Catapano A.L. (2017). Statin use and risk of new-onset diabetes: A meta-analysis of observational studies. Nutr. Metab. Cardiovasc. Dis..

[52] Thakker D., Nair S., Pagada A., Jamdade V., Malik A. (2016). Statin use and the risk of developing diabetes: A network meta-analysis. Pharmacoepidemiol. Drug Saf..

[53] Swerdlow D.I., Preiss D., Kuchenbaecker K.B., Holmes M.V., Engmann J.E.L., Shah T., Sofat R., Stender S., Johnson P.C.D., Scott R.A. (2015). HMG-coenzyme A reductase inhibition, type 2 diabetes, and bodyweight: Evidence from genetic analysis and randomised trials. Lancet.

[54] Fernández-Miranda C., Pérez-Carreras M., Colina F., López-Alonso G., Vargas C., Solís-Herruzo J. (2008). A pilot trial of fenofibrate for the treatment of non-alcoholic fatty liver disease. Dig. Liver Dis..

[55] Kostapanos M.S., Kei A., Elisaf M.S. (2013). Current role of fenofibrate in the prevention and management of non-alcoholic fatty liver disease. World J. Hepatol..

[56] Filippatos T.D., Elisaf M.S. (2011). Role of ezetimibe in non-alcoholic fatty liver disease. World J. Hepatol..

[57] Takeshita Y., Takamura T., Honda M., Kita Y., Zen Y., Kato K.-I., Misu H., Ota T., Nakamura M., Yamada K. (2014). The effects of ezetimibe on non-alcoholic fatty liver disease and glucose metabolism: A randomised controlled trial. Diabetologia.

[58] Lindor K.D., Kowdley K.V., Heathcote E.J., Harrison M.E., Jorgensen R., Angulo P., Lymp J.F., Burgart L., Colin P. (2004). Ursodeoxycholic acid for treatment of nonalcoholic steatohepatitis: Results of a randomized trial. Hepatology.

[59] Dufour J.F., Oneta C.M., Gonvers J.J., Bihl F., Cerny A., Cereda J.M., Zala J.F., Helbling B., Steuerwald M., Zimmermann A. (2006). Randomized placebo-controlled trial of ursodeoxycholic acid with vitamin e in nonalcoholic steatohepatitis. Clin. Gastroenterol. Hepatol..

[60] Van De Wier B., Koek G.H., Bast A., Haenen G.R. (2017). The potential of flavonoids in the treatment of non-alcoholic fatty liver disease. Crit. Rev. Food Sci. Nutr..

[61] Li S., Tan H.Y., Wang N., Cheung F., Hong M., Feng Y. (2018). The Potential and Action Mechanism of Polyphenols in the Treatment of Liver Diseases. Oxid Med. Cell Longev..

[62] Elvira-Torales L.I., García-Alonso J., Periago-Castón M.J. (2019). Nutritional Importance of Carotenoids and Their Effect on Liver Health: A Review. Antioxidants.

[63] De Freitas Carvalho M.M., Lage N.N., de Souza Paulino A.H., Pereira R.R., de Almeida L.T., da Silva T.F., de Brito Magalhães C.L., de Lima W.G., Silva M.E., Pedrosa M.L. (2019). Effects of açai on oxidative stress, ER stress, and inflammation-related parameters in mice with high fat diet-fed induced NAFLD. Sci. Rep..

[64] El Hadi H., Vettor R., Rossato M. (2018). Vitamin E as a Treatment for Nonalcoholic Fatty Liver Disease: Reality or Myth?. Antioxidants.

[65] Colica C., Boccuto L., Abenavoli L. (2017). Silymarin: An option to treat non-alcoholic fatty liver disease. World J. Gastroenterol..

[66] Goh G.B.-B., Pagadala M.R., Dasarathy J., Unalp-Arida A., Sargent R., Hawkins C., Sourianarayanane A., Khiyami A., Yerian L., Pai R.K. (2014). Clinical spectrum of non-alcoholic fatty liver disease in diabetic and non-diabetic patients. BBA Clin..

[67] Laursen T.L., Hagemann C.A., Wei C., Kazankov K., Thomsen K.L., Knop F.K., Grønbæk H. (2019). Bariatric surgery in patients with non-alcoholic fatty liver disease—From pathophysiology to clinical effects. World J. Hepatol..

[68] Vander Naalt S.J., Gurria J.P., Holterman A.L. (2014). Surgical treatment of nonalcoholic fatty liver disease in severely obese patients. Hepat. Med..

[69] Lee Y., Doumouras A.G., Yu J., Brar K., Banfield L., Gmora S., Anvari M., Hong D. (2019). Complete resolution of nonalcoholic fatty liver disease after bariatric surgery: A systematic review and meta-analysis. Clin. Gastroenterol. Hepatol..

[70] Maciejewski M.L., Arterburn D.E. (2013). Cost-effectiveness of bariatric surgery. JAMA.

[71] Bzowej N.H. (2018). Nonalcoholic steatohepatitis: The new frontier for liver transplantation. Curr. Opin. Organ. Transplant..

[72] Neuschwander-Tetri B.A. (2020). Therapeutic landscape for NAFLD in 2020. Gastroenterology.

[73] Wei Q.-Y., He K.-M., Chen J.-L., Xu Y.-M., Lau A.T.Y. (2019). Phytofabrication of Nanoparticles as Novel Drugs for Anticancer Applications. Molecules.

[74] United State National Nanotechnology Institute What is Nanotechnology. https://www.nano.gov/nanotech-101/what/definition.

[75] Krishnaswamy K., Orsat V., Grumezescu A.M. (2017). Chapter 2—Sustainable Delivery Systems through Green Nanotechnology. Nano- and Microscale Drug Delivery Systems.

[76] Zielińska A., Carreiró F., Oliveira A.M., Neves A., Pires B., Venkatesh D.N., Durazzo A., Lucarini M., Eder P., Silva A.M. (2020). Polymeric nanoparticles: Production, characterization, toxicology and ecotoxicology. Molecules.

[77] Lu X.-Y., Wu D.-C., Li Z.-J., Chen G.-Q., Villaverde A. (2011). Chpater 7—Polymer Nanoparticles. Progress in Molecular Biology and Translational Science.

[78] Yoshida T., Lai T.C., Kwon G.S., Sako K. (2013). pH-and ion-sensitive polymers for drug delivery. Exp. Opin. Drug Deliv..

[79] Rana V., Sharma R., Mohapatra S.S., Ranjan S., Dasgupta N., Mishra R.K., Thomas S. (2019). Chapter 5—Recent Advances in Development of Nano Drug Delivery. Applications of Targeted Nano Drugs and Delivery Systems.

[80] Han J., Zhao D., Li D., Wang X., Jin Z., Zhao K. (2018). Polymer-Based Nanomaterials and Applications for Vaccines and Drugs. Polymers.

[81] Jeon S.-Y., Imm J.-Y. (2014). Lipase inhibition and cholesterol-lowering activities of laccase-catalyzed catechin polymers. Food Sci. Biotechnol..

[82] Bruce S.B., Joshua D.B., Zachary O., Bryan M., Mardel C., Kurt M., Kyle P., Jodi H., Lisa-Marie B., Larisa G. (2011). Lipid-Lowering Effects of Polymers Derived from Halophenyl Pyrroles. Lett. Drug Des..

[83] Yang B.K., Park J.B., Song C.H. (2002). Hypolipidemic effect of exo-polymer produced in submerged mycelial culture of five different mushrooms. J. Microb. Biotech..

[84] Alonso L., Calvo M.V., Fontecha J. (2019). The Influence of β-Cyclodextrin on the Reduction of Cholesterol Content in Egg and Duck Liver Pâté. Foods.

[85] Alonso L., Fox P.F., Calvo M.V., Fontecha J. (2018). Effect of Beta Cyclodextrin on the Reduction of Cholesterol in Ewe’s Milk Manchego Cheese. Molecules.

[86] Young I.S., Loughrey B.V., Lip G.Y.H., Hall J.E. (2007). Chapter 88—Lipid-Lowering Therapy. Comprehensive Hypertension.

[87] Korotkova V.D., Perelygina A.A., Lobanova A.M., Stoilov L.D. (1983). Effect of dietary supplements with different cellulose content on the blood glucose and insulin levels in type II diabetes mellitus. Probl. Endokrinol..

[88] Geleva D., Thomas W., Gannon M.C., Keenan J.M. (2003). A Solubilized Cellulose Fiber Decreases Peak Postprandial Cholecystokinin Concentrations after a Liquid Mixed Meal in Hypercholesterolemic Men and Women. J. Nutr..

[89] Andrade D.R.M., Mendonça M.H., Helm C.V., Magalhães W.L.E., de Muniz G.I.B., Kestur S.G. (2015). Assessment of Nano Cellulose from Peach Palm Residue as Potential Food Additive: Part II: Preliminary Studies. J. Food Sci. Technol..

[90] Zhang Q., Yu H., Xiao X., Hu L., Xin F., Yu X. (2018). Inulin-type fructan improves diabetic phenotype and gut microbiota profiles in rats. PeerJ.

[91] Patki A., Shelgaonkar V. (2010). Effect of 6% hydroxyethyl starch-450 and low molecular weight dextran on blood sugar levels during surgery under subarachnoid block: A prospective randomised study. Indian J. Anaesth..

[92] Byrnes S., Brand-Miller J., Denyer G. (1995). Amylopectin starch promotes the development of insulin resistance in rats. J. Nutr..

[93] Babazono T., Nakamoto H., Kasai K., Kuriyama S., Sugimoto T., Nakayama M., Hamada C., Furuya R., Hasegawa H., Kasahara M. (2007). Effects of Icodextrin on Glycemic and Lipid Profiles in Diabetic Patients Undergoing Peritoneal Dialysis. Am. J. Nephrol..

[94] Ramos R., González T., Fulladosa X., Castelao A.M., Grinyó J.M. (2008). False hyperglycemies in diabetic patients using icodextrin in peritoneal dialysis. Dial. Transplant..

[95] US Food and Drug Administration, FDA. 2012 (2013). GRAS Notices: Shrimp-Derived Chitosan. https://www.cfsanappsexternal.fda.gov/scripts/fdcc/index.cfm?set=GRASNotices&id=443&sort=GRN_No&order=DESC&startrow=1&type=basic&search=chitosan.

[96] Ozcelik E., Uslu S., Erkasap N., Karimi H. (2014). Protective effect of chitosan treatment against acetaminophen-induced hepatotoxicity. Kaohsiung J. Med. Sci..

[97] Liang J., Liu Y., Liu J., Li Z., Fan Q., Jiang Z., Yan F., Wang Z., Huang P., Feng N. (2018). Chitosan-functionalized lipid-polymer hybrid nanoparticles for oral delivery of silymarin and enhanced lipid-lowering effect in NAFLD. J. Nanobiotechnol..

[98] Liu S.H., Chiu C.Y., Shi C.M., Chiang M.T. (2018). Functional Comparison of High and Low Molecular Weight Chitosan on Lipid Metabolism and Signals in High-Fat Diet-Fed Rats. Mar. Drugs.

[99] Wang Z.F., Wang M.Y., Yu D.H., Zhao Y., Xu H.M., Zhong S., Sun W.Y., He Y.F., Niu J.Q., Gao P.J. (2018). Therapeutic effect of chitosan on CCl4-induced hepatic fibrosis in rats. Mol. Med. Rep..

[100] Prajapati B. (2009). Chitosan a marine medical polymer and its lipid lowering capacity. Int. J. Health.

[101] Cheng M., Zhu W., Li Q., Dai D., Hou Y. (2017). Anti-cancer efficacy of biotinylated chitosan nanoparticles in liver cancer. Oncotarget.

[102] Bonferoni M.C., Gavini E., Rassu G., Maestri M., Giunchedi P. (2020). Chitosan Nanoparticles for Therapy and Theranostics of Hepatocellular Carcinoma (HCC) and Liver-Targeting. Nanomaterials.

[103] Na J.H., Koo H., Lee S., Han S.J., Lee K.E., Kim S., Lee H., Lee S., Choi K., Kwon I.C. (2016). Precise Targeting of Liver Tumor Using Glycol Chitosan Nanoparticles: Mechanisms, Key Factors, and Their Implications. Mol. Pharm..

[104] Abd-Allah H., Nasr M., Ahmed-Farid O.A.H., Ibrahim B.M.M., Bakeer R.M., Ahmed R.F. (2020). Nicotinamide and ascorbic acid nanoparticles against the hepatic insult induced in rats by high fat high fructose diet: A comparative study. Life Sci..

[105] Makadia H.K., Siegel S.J. (2011). Poly Lactic-co-Glycolic Acid (PLGA) as Biodegradable Controlled Drug Delivery Carrier. Polymers.

[106] Morelli L., Gimondi S., Sevieri M., Salvioni L., Guizzetti M., Colzani B., Palugan L., Foppoli A., Talamini L., Morosi L. (2019). Monitoring the Fate of Orally Administered PLGA Nanoformulation for Local Delivery of Therapeutic Drugs. Pharmaceutics.

[107] Jazayeri-Tehrani S.A., Rezayat S.M., Mansouri S., Qorbani M., Alavian S.M., Daneshi-Maskooni M., Hosseinzadeh-Attar M.-J. (2019). Nano-curcumin improves glucose indices, lipids, inflammation, and Nesfatin in overweight and obese patients with non-alcoholic fatty liver disease (NAFLD): A double-blind randomized placebo-controlled clinical trial. Nutr. Metab..

[108] Jazayeri-Tehrani S.A., Rezayat S.M., Mansouri S., Qorbani M., Alavian S.M., Daneshi-Maskooni M., Hosseinzadeh-Attar M.-J. (2017). Efficacy of nanocurcumin supplementation on insulin resistance, lipids, inflammatory factors and nesfatin among obese patients with non-alcoholic fatty liver disease (NAFLD): A trial protocol. BMJ Open.

[109] Sasaki H., Sunagawa Y., Takahashi K., Imaizumi A., Fukuda H., Hashimoto T., Wada H., Katanasaka Y., Kakeya H., Fujita M. (2011). Innovative Preparation of Curcumin for Improved Oral Bioavailability. Biol. Pharm. Bull..

[110] Abenavoli L., Milic N., Luzza F., Boccuto L., De Lorenzo A. (2017). Polyphenols Treatment in Patients with Nonalcoholic Fatty Liver Disease. J. Transl. Int. Med..

[111] Wan S., Zhang L., Quan Y., Wei K. (2018). Resveratrol-loaded PLGA nanoparticles: Enhanced stability, solubility and bioactivity of resveratrol for non-alcoholic fatty liver disease therapy. R. Soc. Open Sci..

[112] He S., Guo W., Deng F., Chen K., Jiang Y., Dong M., Peng L., Chen X. (2018). Targeted delivery of microRNA 146b mimic to hepatocytes by lactosylated PDMAEMA nanoparticles for the treatment of NAFLD. Artif. Cells Nanomed. Biotechnol..

[113] Boey A., Ho H.K. (2020). All Roads Lead to the Liver: Metal Nanoparticles and Their Implications for Liver Health. Small.

[114] Crans S.T.D.C. (2018). Metal Nanoparticles in Nanomedicine: Advantages and Scope. Metal Nanoparticles.

[115] Venkatesh N., Bhowmik H., Kuila A. (2018). Metallic nanoparticle: A review. Biomed. J. Sci. Tech. Res..

[116] Mody V.V., Siwale R., Singh A., Mody H.R. (2010). Introduction to metallic nanoparticles. J. Pharm. Bioallied Sci..

[117] Khan I., Saeed K., Khan I. (2019). Nanoparticles: Properties, applications and toxicities. Arab. J. Chem..

[118] Golbamaki N., Rasulev B., Cassano A., Marchese Robinson R.L., Benfenati E., Leszczynski J., Cronin M.T.D. (2015). Correction: Genotoxicity of metal oxide nanomaterials: Review of recent data and discussion of possible mechanisms. Nanoscale.

[119] Peng F., Tee J.K., Setyawati M.I., Ding X., Yeo H.L.A., Tan Y.L., Leong D.T., Ho H.K. (2018). Inorganic Nanomaterials as Highly Efficient Inhibitors of Cellular Hepatic Fibrosis. ACS Appl. Mater. Interfaces.

[120] Ibrahim K.E., Al-Mutary M.G., Bakhiet A.O., Khan H.A. (2018). Histopathology of the liver, kidney, and spleen of mice exposed to gold nanoparticles. Molecules.

[121] Carvajal S., Perramón M., Oró D., Casals E., Fernández-Varo G., Casals G., Parra M., de la Presa B.G., Ribera J., Pastor Ó. (2019). Cerium oxide nanoparticles display antilipogenic effect in rats with non-alcoholic fatty liver disease. Sci. Rep..

[122] Oró D., Yudina T., Fernández-Varo G., Casals E., Reichenbach V., Casals G., de la Presa B.G., Sandalinas S., Carvajal S., Puntes V. (2016). Cerium oxide nanoparticles reduce steatosis, portal hypertension and display anti-inflammatory properties in rats with liver fibrosis. J. Hepatol..

[123] Parra-Robert M., Casals E., Massana N., Zeng M., Perramón M., Fernández-Varo G., Morales-Ruiz M., Puntes V., Jiménez W., Casals G. (2019). Beyond the Scavenging of Reactive Oxygen Species (ROS): Direct Effect of Cerium Oxide Nanoparticles in Reducing Fatty Acids Content in an In Vitro Model of Hepatocellular Steatosis. Biomolecules.

[124] Sun X., Liu Z., Welsher K., Robinson J.T., Goodwin A., Zaric S., Dai H. (2008). Nano-graphene oxide for cellular imaging and drug delivery. Nano Res..

[125] Tabish T.A. (2018). Graphene-based materials: The missing piece in nanomedicine? Biochem. Biophys. Res. Commun..

[126] Reina G., González-Domínguez J.M., Criado A., Vázquez E., Bianco A., Prato M. (2017). Promises, facts and challenges for graphene in biomedical applications. Chem. Soc. Rev..

[127] De Marchi L., Pretti C., Gabriel B., Marques P.A., Freitas R., Neto V. (2018). An overview of graphene materials: Properties, applications and toxicity on aquatic environments. Sci. Total Environ..

[128] Gai S., Yang G., Yang P., He F., Lin J., Jin D., Xing B. (2018). Recent advances in functional nanomaterials for light–triggered cancer therapy. Nano Today.

[129] Enoki T., Prasad B.L.V., Shibayama Y., Takai K., Sato H., Yasuda E.-I., Inagaki M., Kaneko K., Endo M., Oya A., Tanabe Y. (2003). Chapter 23—Magnetism of Nano-graphite. Carbon Alloys.

[130] Amaro-Gahete J., Benítez A., Otero R., Esquivel D., Jiménez-Sanchidrián C., Morales J., Caballero Á., Romero-Salguero F.J. (2019). A Comparative Study of Particle Size Distribution of Graphene Nanosheets Synthesized by an Ultrasound-Assisted Method. Nanomaterials.

[131] Pei X., Zhu Z., Gan Z., Chen J., Zhang X., Cheng X., Wan Q., Wang J. (2020). PEGylated nano-graphene oxide as a nanocarrier for delivering mixed anticancer drugs to improve anticancer activity. Sci. Rep..

[132] Masoudipour E., Kashanian S., Maleki N. (2017). A targeted drug delivery system based on dopamine functionalized nano graphene oxide. Chem. Phys. Lett..

[133] Smith A.T., LaChance A.M., Zeng S., Liu B., Sun L. (2019). Synthesis, properties, and applications of graphene oxide/reduced graphene oxide and their nanocomposites. Nano Mater. Sci..

[134] Firdhouse M.J., Lalitha P. (2016). Nanosilver-decorated nanographene and their adsorption performance in waste water treatment. Bioresour Bioprocess.

[135] Majid R., Pegah M., Hadis L., Behzad B., Amirhossein S. (2020). Conjugates of Curcumin with Graphene and Carbon Nanotubes: A Review on Biomedical Applications. Curr. Med..

[136] Mugnano M., Lama G.C., Castaldo R., Marchesano V., Merola F., del Giudice D., Calabuig A., Gentile G., Ambrogi V., Cerruti P. (2020). Cellular Uptake of Mildly Oxidized Nanographene for Drug-Delivery Applications. ACS Appl. Nano Mater..

[137] Dasari Shareena T.P., McShan D., Dasmahapatra A.K., Tchounwou P.B. (2018). A Review on Graphene-Based Nanomaterials in Biomedical Applications and Risks in Environment and Health. Nano-Micro Lett..

[138] Nurunnabi M., Parvez K., Nafiujjaman M., Revuri V., Khan H.A., Feng X., Lee Y.-K. (2015). Bioapplication of graphene oxide derivatives: Drug/gene delivery, imaging, polymeric modification, toxicology, therapeutics and challenges. RSC Adv..

[139] Ou L., Song B., Liang H., Liu J., Feng X., Deng B., Sun T., Shao L. (2016). Toxicity of graphene-family nanoparticles: A general review of the origins and mechanisms. Part. Fibre Toxicol..

[140] Cai J., Gou X., Sun B., Li W., Li D., Liu J., Hu F., Li Y. (2019). Porous graphene-black phosphorus nanocomposite modified electrode for detection of leptin. Biosens. Bioelectron..

[141] Gong X., Liu Y., Xiang H., Liu H., Liu Z., Zhao X., Li J., Li H., Hong G., Hu T.S. (2019). Membraneless reproducible MoS2 field-effect transistor biosensor for high sensitive and selective detection of FGF21. Sci. China Mater..

[142] Hsueh Y.-H., Hsieh C.-T., Chiu S.-T., Tsai P.-H., Liu C.-Y., Ke W.-J. (2019). Antibacterial Property of Composites of Reduced Graphene Oxide with Nano-Silver and Zinc Oxide Nanoparticles Synthesized Using a Microwave-Assisted Approach. Int. J. Mol. Sci..

[143] Bangham A., Standish M.M., Watkins J.C. (1965). Diffusion of univalent ions across the lamellae of swollen phospholipids. J. Mol. Bio..

[144] Akbarzadeh A., Rezaei-Sadabady R., Davaran S., Joo S.W., Zarghami N., Hanifehpour Y., Samiei M., Kouhi M., Nejati-Koshki K. (2013). Liposome: Classification, preparation, and applications. Nanoscale Res. Lett..

[145] Mishra N., Yadav N.P., Rai V.K., Sinha P., Yadav K.S., Jain S., Arora S. (2013). Efficient Hepatic Delivery of Drugs: Novel Strategies and Their Significance. BioMed Res. Int..

[146] Himanshu A., Sitasharan P., Singhai A. (2011). Liposomes as drug carriers. IJPLS.

[147] Riaz M. (1996). Liposomes preparation methods. Pak. J. Pharm. Sci..

[148] Jiang H., Li Z.-P., Tian G.-X., Pan R.-Y., Xu C.-M., Zhang B., Wu J.-L. (2019). Liver-targeted liposomes for codelivery of curcumin and combretastatin A4 phosphate: Preparation, characterization, and antitumor effects. Int. J. Nanomed..

[149] Li W.-J., Lian Y.-W., Guan Q.-S., Li N., Liang W.-J., Liu W.-X., Huang Y.-B., Cheng Y., Luo H. (2018). Liver-targeted delivery of liposome-encapsulated curcumol using galactosylated-stearate. Exp. Ther. Med..

[150] Sercombe L., Veerati T., Moheimani F., Wu S.Y., Sood A.K., Hua S. (2015). Advances and Challenges of Liposome Assisted Drug Delivery. Front. Pharmacol..

[151] Cao Y., Xu L., Chen C., Wang Y., Zhang Q., Qi R. (2016). Fenofibrate nanoliposome: Preparation and its inhibitory effects on nonalcoholic fatty liver disease in mice. Nanomedicine.

[152] Lee M.-K. (2020). Liposomes for Enhanced Bioavailability of Water-Insoluble Drugs: In Vivo Evidence and Recent Approaches. Pharmaceutics.

[153] Chen C., Jie X., Ou Y., Cao Y., Xu L., Wang Y., Qi R. (2017). Nanoliposome improves inhibitory effects of naringenin on nonalcoholic fatty liver disease in mice. Nanomedicine.

[154] Singh A., Verma N., Kumar K., Grumezescu V., Grumezescu A.M. (2019). Chapter 2—Hybrid composites: A revolutionary trend in biomedical engineering. Materials for Biomedical Engineering.

[155] Narvekar M., Xue H.Y., Wong H.L. (2012). A novel hybrid delivery system: Polymer-oil nanostructured carrier for controlled delivery of highly lipophilic drug all-trans-retinoic acid (ATRA). Int. J. Pharm..

[156] Narvekar M., Xue H.Y., Tran N.T., Mikhael M., Wong H.L. (2014). A new nanostructured carrier design including oil to enhance the pharmaceutical properties of retinoid therapy and its therapeutic effects on chemo-resistant ovarian cancer. Eur. J. Pharm. Biopharm..

[157] Ghitman J., Biru E.I., Stan R., Iovu H. (2020). Review of hybrid PLGA nanoparticles: Future of smart drug delivery and theranostics medicine. Mater. Des..

[158] Mukherjee A., Waters A.K., Kalyan P., Achrol A.S., Kesari S., Yenugonda V.M. (2019). Lipid-polymer hybrid nanoparticles as a next-generation drug delivery platform: State of the art, emerging technologies, and perspectives. Int. J. Nanomed..

[159] Dave V., Yadav R.B., Kushwaha K., Yadav S., Sharma S., Agrawal U. (2017). Lipid-polymer hybrid nanoparticles: Development & statistical optimization of norfloxacin for topical drug delivery system. Bioact. Mater..

[160] Fang R.H., Aryal S., Hu C.M., Zhang L. (2010). Quick synthesis of lipid-polymer hybrid nanoparticles with low polydispersity using a single-step sonication method. Langmuir.

[161] Kim Y., Lee Chung B., Ma M., Mulder W.J., Fayad Z.A., Farokhzad O.C., Langer R. (2012). Mass production and size control of lipid-polymer hybrid nanoparticles through controlled microvortices. Nano Lett..

[162] Sah H., Thoma L.A., Desu H.R., Sah E., Wood G.C. (2013). Concepts and practices used to develop functional PLGA-based nanoparticulate systems. Int. J. Nanomed..

[163] Tan S., Li X., Guo Y., Zhang Z. (2013). Lipid-enveloped hybrid nanoparticles for drug delivery. Nanoscale.

[164] Kamaly N., Yameen B., Wu J., Farokhzad O.C. (2016). Degradable controlled-release polymers and polymeric nanoparticles: Mechanisms of controlling drug release. Chem. Rev..

[165] Hadinoto K., Sundaresan A., Cheow W.S. (2013). Lipid–polymer hybrid nanoparticles as a new generation therapeutic delivery platform: A review. Eur. J. Pharm. Biopharm..

[166] Chabok A., Shamsipur M., Yeganeh-Faal A., Molaabasi F., Molaei K., Sarparast M. (2019). A highly selective semiconducting polymer dots-based “off–on” fluorescent nanoprobe for iron, copper and histidine detection and imaging in living cells. Talanta.

[167] Perni S., Prokopovich P. (2017). Poly-beta-amino-esters nano-vehicles based drug delivery system for cartilage. Nanomedicine.

[168] Ishak R.A.H., Mostafa N.M., Kamel A.O. (2017). Stealth lipid polymer hybrid nanoparticles loaded with rutin for effective brain delivery—Comparative study with the gold standard (Tween 80): Optimization, characterization and biodistribution. Drug Deliv..

[169] Liu Y., Zhao Y., Liu J., Zhang M., Yu M., Feng N. (2016). Wheat germ agglutinin modification of lipid–polymer hybrid nanoparticles: Enhanced cellular uptake and bioadhesion. RSC Adv..

[170] Mohanty A., Uthaman S., Park I.-K. (2020). Utilization of Polymer-Lipid Hybrid Nanoparticles for Targeted Anti-Cancer Therapy. Molecules.

[171] Sajid M., Cameotra S.S., Ahmad Khan M.S., Ahmad I., Ahmad Khan M.S., Ahmad I., Chattopadhyay D. (2019). Chapter 23—Nanoparticle-Based Delivery of Phytomedicines: Challenges and Opportunities. New Look to Phytomedicine.

[172] Borges A., Freitas V.D., Mateus N., Fernandes I., Oliveira J. (2020). Solid Lipid Nanoparticles as Carriers of Natural Phenolic Compounds. Antioxidants.

[173] Shah R., Eldridge D., Palombo E., Harding I. (2015). Composition and structure. Lipid Nanoparticles: Production, Characterization and Stability.

[174] Fonseca-Santos B., Gremião M.P.D., Chorilli M. (2015). Nanotechnology-based drug delivery systems for the treatment of Alzheimer’s disease. Int. J. Nanomed..

[175] Badrealam K.F., Owais M. (2014). Nanoscale drug delivery systems: An updated view. Nanobiotechnology.

[176] Duan Y., Dhar A., Patel C., Khimani M., Neogi S., Sharma P., Siva Kumar N., Vekariya R.L. (2020). A brief review on solid lipid nanoparticles: Part and parcel of contemporary drug delivery systems. RSC Adv..

[177] Nayak A.P., Tiyaboonchai W., Patankar S., Madhusudhan B., Souto E.B. (2010). Curcuminoids-loaded lipid nanoparticles: Novel approach towards malaria treatment. Colloids Surf. B.

[178] Mukherjee S., Ray S., Thakur R.S. (2009). Solid lipid nanoparticles: A modern formulation approach in drug delivery system. Indian J. Pharm. Sci..

[179] Battaglia L., Gallarate M., Panciani P.P., Ugazio E., Sapino S., Peira E., Chirio D. (2015). Techniques for the Preparation of Solid Lipid Nano and Microparticles.

[180] Naseri N., Valizadeh H., Zakeri-Milani P. (2015). Solid Lipid Nanoparticles and Nanostructured Lipid Carriers: Structure, Preparation and Application. Adv. Pharm. Bull..

[181] Battu S.K., Repka M.A., Maddineni S., Chittiboyina A.G., Avery M.A., Majumdar S. (2010). Physicochemical characterization of berberine chloride: A perspective in the development of a solution dosage form for oral delivery. AAPS PharmSciTech.

[182] Xue M., Yang M.-X., Zhang W., Li X.-M., Gao D.-H., Ou Z.-M., Li Z.-P., Liu S.-H., Li X.-J., Yang S.-Y. (2013). Characterization, pharmacokinetics, and hypoglycemic effect of berberine loaded solid lipid nanoparticles. Int. J. Nanomed..

[183] Xue M., Zhang L., Yang M.-X., Zhang W., Li X.-M., Ou Z.-M., Li Z.-P., Liu S.-H., Li X.-J., Yang S.-Y. (2015). Berberine-loaded solid lipid nanoparticles are concentrated in the liver and ameliorate hepatosteatosis in db/db mice. Int. J. Nanomed..

[184] Khan S., Baboota S., Ali J., Khan S., Narang R.S., Narang J.K. (2015). Nanostructured lipid carriers: An emerging platform for improving oral bioavailability of lipophilic drugs. Int. J. Pharm. Investig..

[185] Singh B., Beg S., Khurana R.K., Sandhu P.S., Kaur R., Katare O.P. (2014). Recent advances in self-emulsifying drug delivery systems (SEDDS). Crit. Rev. Ther. Drug Carrier Syst..

[186] Zhang L., Zhang L., Zhang M., Pang Y., Li Z., Zhao A., Feng J. (2015). Self-emulsifying drug delivery system and the applications in herbal drugs. Drug Deliv..

[187] Salimi A., Sharif Makhmal Zadeh B., Hemati A.A., Akbari Birgani S. (2014). Design and Evaluation of Self-Emulsifying Drug Delivery System (SEDDS) Of Carvedilol to Improve the Oral Absorption. Jundishapur. J. Nat. Pharm. Prod..

[188] Akula S., Gurram A.K., Devireddy S.R. (2014). Self-Microemulsifying Drug Delivery Systems: An Attractive Strategy for Enhanced Therapeutic Profile. Int. Sch. Res. Not..

[189] Pouton C.W. (2000). Lipid formulations for oral administration of drugs: Non-emulsifying, self-emulsifying and ‘self-microemulsifying’ drug delivery systems. Eur. J. Pharm. Sci..

[190] Pouton C.W. (2006). Formulation of poorly water-soluble drugs for oral administration: Physicochemical and physiological issues and the lipid formulation classification system. Eur. J. Pharm. Sci..

[191] Eltobshi A.A., Mohamed E.A., Abdelghani G.M., Nouh A.T. (2018). Self-nanoemulsifying drug-delivery systems for potentiated anti-inflammatory activity of diacerein. Int. J. Nanomed..

[192] Gardouh A.R., Nasef A.M., Mostafa Y., Gad S. (2020). Design and evaluation of combined atorvastatin and ezetimibe optimized self- nano emulsifying drug delivery system. J. Drug Deliv. Sci. Technol..

[193] Nikolakakis I., Partheniadis I. (2017). Self-emulsifying granules and pellets: Composition and formation mechanisms for instant or controlled release. Pharmaceutics.

[194] Mohd Izham M.N., Hussin Y., Aziz M.N.M., Yeap S.K., Rahman H.S., Masarudin M.J., Mohamad N.E., Abdullah R., Alitheen N.B. (2019). Preparation and characterization of self nano-emulsifying drug delivery system loaded with citraland its antiproliferative effect on colorectal cells in vitro. Nanomaterials.

[195] Abou Assi R., Abdulbaqi M.I., Seok Ming T., Siok Yee C., Wahab A.H., Asif S.M., Darwis Y. (2020). Liquid and Solid Self-Emulsifying Drug Delivery Systems (SEDDs) as Carriers for the Oral Delivery of Azithromycin: Optimization, In Vitro Characterization and Stability Assessment. Pharmaceutics.

[196] Gershanik T., Benita S. (1996). Positively charged self-emulsifying oil formulation for improving oral bioavailability of progesterone. Pharm. Dev. Technol..

[197] Malkawi A., Jalil A., Nazir I., Matuszczak B., Kennedy R., Bernkop-Schnürch A. (2020). Self-Emulsifying Drug Delivery Systems: Hydrophobic Drug Polymer Complexes Provide a Sustained Release in Vitro. Mol. Pharm..

[198] Piyush Chudiwal S. (2018). Solid self-microemulsifying drug delivery system (SMEDDS) of primaquine: Bio-distribution and enhanced liver uptake. J. Nanomed. Nanotechnol..

[199] Rajpoot K., Tekade M., Pandey V., Nagaraja S., Youngren-Ortiz S.R., Tekade R.K., Tekade R.K. (2020). Chapter 9—Self-microemulsifying drug-delivery system: Ongoing challenges and future ahead. Drug Delivery Systems.

[200] Aristote B., Buya A.B., Patrick B. (2020). Memvanga and Véronique Préat. Self-Nano-Emulsifying Drug-Delivery Systems: From the Development to the Current Applications and Challenges in Oral Drug Delivery. Pharmaceutics.

[201] Mehnert W., Mäder K. (2001). Solid Lipid Nanoparticles: Production, Characterization and Applications. Adv. Drug Deliv. Rev..

[202] Müller R.H., Radtke M., Wissing S.A. (2002). Solid lipid nanoparticles (SLN) and nanostructured lipid carriers (NLC) in cosmetic and dermatological preparations. Adv. Drug Deliv. Rev..

[203] Müller R., Radtke M., Wissing S. (2002). Nanostructured lipid matrices for improved microencapsulation of drugs. Int. J. Pharm..

[204] Khosa A., Reddi S., Saha R.N. (2018). Nanostructured lipid carriers for site-specific drug delivery. Biomed. Pharmacother..

[205] Uprit S., Kumar Sahu R., Roy A., Pare A. (2013). Preparation and characterization of minoxidil loaded nanostructured lipid carrier gel for effective treatment of alopecia. Saudi Pharm. J..

[206] Subramaniam B., Siddik Z.H., Nagoor N.H. (2020). Optimization of nanostructured lipid carriers: Understanding the types, designs, and parameters in the process of formulations. J. Nanopart Res..

[207] Chauhan I., Yasir M., Verma M., Singh A.P. (2020). Nanostructured Lipid Carriers: A Groundbreaking Approach for Transdermal Drug Delivery. Adv. Pharm. Bull..

[208] Ding X., Xu X., Zhao Y., Zhang L., Yu Y., Huang F., Yin D., Huang H. (2017). Tumor targeted nanostructured lipid carrier co-delivering paclitaxel and indocyanine green for laser triggered synergetic therapy of cancer. Rsc Adv..

[209] Feng F., Zheng D., Zhang D., Duan C., Wang Y., Jia L., Wang F., Liu Y., Gao Q., Zhang Q. (2011). Preparation, characterization and biodistribution of nanostructured lipid carriers for parenteral delivery of bifendate. J. Microencapsul..

[210] Chen C.H., Chen C.J., Elzoghby A.O., Yeh T.S., Fang J.Y. (2018). Self-assembly and directed assembly of lipid nanocarriers for prevention of liver fibrosis in obese rats: A comparison with the therapy of bariatric surgery. Nanomedicine.

[211] Wang Q., Ou Y., Hu G., Wen C., Yue S., Chen C., Xu L., Xie J., Dai H., Xiao H. (2020). Naringenin attenuates non-alcoholic fatty liver disease by down-regulating the NLRP3/NF-κB pathway in mice. Br. J. Pharmacol..

[212] Hu R., Liu S., Anwaier G., Wang Q., Shen W., Shen Q., Qi R. (2021). Formulation and intestinal absorption of naringenin loaded nanostructured lipid carrier and its inhibitory effects on nonalcoholic fatty liver disease. Nanomedicine.

[213] Kumar M., Bishnoi R.S., Shukla A.K., Jain C.P. (2019). Techniques for Formulation of Nanoemulsion Drug Delivery System: A Review. Prev. Nutr. Food Sci..

[214] Aboalnaja K.O., Yaghmoor S., Kumosani T.A., McClements D.J. (2016). Utilization of nanoemulsions to enhance bioactivity of pharmaceuticals, supplements, and nutraceuticals: Nanoemulsion delivery systems and nanoemulsion excipient systems. Exp. Opin. Drug Deliv..

[215] Choudhury H., Gorain B., Karmakar S., Biswas E., Dey G., Barik R., Mandal M., Pal T.K. (2014). Improvement of cellular uptake, in vitro antitumor activity and sustained release profile with increased bioavailability from a nanoemulsion platform. Int. J. Pharm..

[216] Kumar N., Das B., Patra S., Grumezescu A.M. (2017). Chapter 10—Drug Resistance in Tuberculosis: Nanomedicines at Rescue. Antimicrobial Nanoarchitectonics.

[217] Laxmi M., Bhardwaj A., Mehta S., Mehta A. (2015). Development and characterization of nanoemulsion as carrier for the enhancement of bioavailability of artemether. Artif. Cells Nanomed. Biotechnol..

[218] Sharma B., Sharma A., Arora S., Gupta S., Bishnoi M. (2012). Formulation, optimization and evaluation of atorvastatin calcium loaded microemulsion. J. Pharm. Drug Deliv. Res..

[219] Ali H.H., Hussein A.A. (2017). Oral nanoemulsions of candesartan cilexetil: Formulation, characterization and in vitro drug release studies. AAPS Open.

[220] Jiang T., Liao W., Charcosset C. (2020). Recent advances in encapsulation of curcumin in nanoemulsions: A review of encapsulation technologies, bioaccessibility and applications. Food Res. Int..

[221] Sarker D.K. (2005). Engineering of nanoemulsions for drug delivery. Curr. Drug Deliv..

[222] Yoon H.J., Zhang X., Kang M.G., Kim G.J., Shin S.Y., Baek S.H., Lee B.N., Hong S.J., Kim J.T., Hong K. (2018). Cytotoxicity evaluation of turmeric extract incorporated oil-in-water nanoemulsion. Int. J. Mol. Sci..

[223] Aditya N.P., Macedo A.S., Doktorovova S., Souto E.B., Kim S., Chang P.-S., Ko S. (2014). Development and evaluation of lipid nanocarriers for quercetin delivery: A comparative study of solid lipid nanoparticles (SLN), nanostructured lipid carriers (NLC), and lipid nanoemulsions (LNE). LWT Food Sci. Technol..

[224] Gönüllü Ü., Üner M., Yener G., Karaman E.F., Aydoğmuş Z. (2015). Formulation and characterization of solid lipid nanoparticles, nanostructured lipid carriers and nanoemulsion of lornoxicam for transdermal delivery. Acta Pharm..

[225] Puglia C., Damiani E., Offerta A., Rizza L., Tirendi G.G., Tarico M.S., Curreri S., Bonina F., Perrotta R.E. (2014). Evaluation of nanostructured lipid carriers (NLC) and nanoemulsions as carriers for UV-filters: Characterization, in vitro penetration and photostability studies. Eur. J. Pharm. Sci..

[226] Shakeel F., Wafa R., Shafiq S. (2009). Solubility and Dissolution Improvement of Aceclofenac using Different Nanocarriers. J. Bioequivalence Bioavailab..

[227] Dammak I., Sobral P.J.D.A., Aquino A., Neves M.A.D., Conte-Junior C.A. (2020). Nanoemulsions: Using emulsifiers from natural sources replacing synthetic ones—A review. Compr. Rev. Food Sci. Food Saf..

[228] Norouzi P., Rastegari A., Mottaghitalab F., Farokhi M., Zarrintaj P., Saeb M.R., Mozafari M. (2020). 24—Nanoemulsions for intravenous drug delivery. Nanoengineered Biomaterials for Advanced Drug Delivery.

[229] Ye H., He X., Feng X. (2020). Developing neobavaisoflavone nanoemulsion suppresses lung cancer progression by regulating tumor microenvironment. Biomed. Pharmacother..

[230] Singh K.K., Vingkar S.K. (2008). Formulation, antimalarial activity and biodistribution of oral lipid nanoemulsion of primaquine. Int. J. Pharm..

[231] Simion V., Stan D., Constantinescu C.A., Deleanu M., Dragan E., Tucureanu M.M., Gan A.-M., Butoi E., Constantin A., Manduteanu I. (2016). Conjugation of curcumin-loaded lipid nanoemulsions with cell-penetrating peptides increases their cellular uptake and enhances the anti-inflammatory effects in endothelial cells. J. Pharm. Pharmacol..

[232] Dong D., Quan E., Yuan X., Xie Q., Li Z., Wu B. (2017). Sodium Oleate-Based Nanoemulsion Enhances Oral Absorption of Chrysin through Inhibition of UGT-Mediated Metabolism. Mol. Pharm..

[233] Jiang S. (2015). Enhanced Physicochemical and Functional Properties of Pea (Pisum sativum) Protein by pH-Shifting and Ultrasonication Combined Process.

[234] El-Sherbiny M., Eldosoky M., El-Shafey M., Othman G., Elkattawy H.A., Bedir T., Elsherbiny N.M. (2018). Vitamin D nanoemulsion enhances hepatoprotective effect of conventional vitamin D in rats fed with a high-fat diet. Chem. Biol. Interact..

[235] Trivedi R., Kompella U.B. (2010). Nanomicellar formulations for sustained drug delivery: Strategies and underlying principles. Nanomedicine.

[236] Velagaleti P., Anglade E., Khan I., Gilger B., Mitra A. (2010). Topical delivery of hydrophobic drugs using a novel mixed nanomicellar technology to treat diseases of the anterior and posterior segments of the eye. Drug Deliv. Technol..

[237] Determan M.D., Cox J.P., Mallapragada S.K. (2007). Drug release from pH-responsive thermogelling pentablock copolymers. J. Biomed. Mater. Res. A.

[238] Qiu L., Zhang J., Yan M., Jin Y., Zhu K. (2007). Reverse self-assemblies based on amphiphilic polyphosphazenes for encapsulation of water-soluble molecules. Nanotechnology.

[239] Wu Y.-L., Li Z. (2019). The perspectives of using unimolecular micelles in nanodrug formulation. Ther. Deliv..

[240] Vadlapudi A.D., Mitra A.K. (2013). Nanomicelles: An emerging platform for drug delivery to the eye. Ther. Deliv..

[241] Nishiyama N., Kataoka K. (2006). Current state, achievements, and future prospects of polymeric micelles as nanocarriers for drug and gene delivery. Pharmacol. Ther..

[242] Wang Z., Deng X., Ding J., Zhou W., Zheng X., Tang G. (2018). Mechanisms of drug release in pH-sensitive micelles for tumour targeted drug delivery system: A review. Int. J. Pharm..

[243] Torchilin V.P. (2007). Micellar nanocarriers: Pharmaceutical perspectives. Pharm. Res..

[244] Kulthe S.S., Choudhari Y.M., Inamdar N.N., Mourya V. (2012). Polymeric micelles: Authoritative aspects for drug delivery. Des. Monomers Polym..

[245] Hussein Y.H., Youssry M. (2018). Polymeric micelles of biodegradable diblock copolymers: Enhanced encapsulation of hydrophobic drugs. Materials.

[246] Zhang Z., Yang L., Hou J., Xia X., Wang J., Ning Q., Jiang S. (2018). Promising positive liver targeting delivery system based on arabinogalactan-anchored polymeric micelles of norcantharidin. Artif. Cells Nanomed. Biotechnol..

[247] Avramović N., Mandić B., Savić-Radojević A., Simić T. (2020). Polymeric Nanocarriers of Drug Delivery Systems in Cancer Therapy. Pharmaceutics.

[248] Li P., Han J., Li D., Chen J., Wang W., Xu W. (2018). Synthetic glycopolypeptide micelle for targeted drug delivery to hepatic carcinoma. Polymers.

[249] Li Z.P., Tian G.X., Jiang H., Pan R.Y., Lian B., Wang M., Gao Z.Q., Zhang B., Wu J.L. (2019). Liver-Targeting and pH-Sensitive Sulfated Hyaluronic Acid Mixed Micelles for Hepatoma Therapy. Int. J. Nanomed..

[250] Kumar V., Mondal G., Dutta R., Mahato R.I. (2016). Co-delivery of small molecule hedgehog inhibitor and miRNA for treating liver fibrosis. Biomaterials.

[251] Mohammad I.S., Hu H., Yin L., He W. (2019). Drug nanocrystals: Fabrication methods and promising therapeutic applications. Int. J. Pharm..

[252] Chang T.-L., Zhan H., Liang D., Liang J.F. (2015). Nanocrystal technology for drug formulation and delivery. Front. Chem. Sci. Eng..

[253] Junyaprasert V.B., Morakul B. (2015). Nanocrystals for enhancement of oral bioavailability of poorly water-soluble drugs. Asian J. Pharm. Sci..

[254] Ahn H.-Y., Lee H.-E., Jin K., Nam K.T. (2013). Extended gold nano-morphology diagram: Synthesis of rhombic dodecahedra using CTAB and ascorbic acid. J. Mater. Chem. C.

[255] Lipinski C. (2002). Poor aqueous solubility—an industry wide problem in drug discovery. Am. Pharm. Rev..

[256] Couvreur P., Tulkenst P., Roland M., Trouet A., Speiser P. (1977). Nanocapsules: A new type of lysosomotropic carrier. FEBS Lett..

[257] Crommelin D.J.A., Storm G. (2003). Liposomes: From the Bench to the Bed. J. Liposome Res..

[258] Muller H.R., Shegokar R., Keck M.C. (2011). 20 years of lipid nanoparticles (SLN & NLC): Present state of development & industrial applications. Curr. Drug Discov. Technol..

[259] Singh Y., Meher J.G., Raval K., Khan F.A., Chaurasia M., Jain N.K., Chourasia M.K. (2017). Nanoemulsion: Concepts, development and applications in drug delivery. J. Control. Release.

[260] Salazar J., Müller R.H., Möschwitzer J.P. (2014). Combinative particle size reduction technologies for the production of drug nanocrystals. J. Pharm..

[261] Chan H.-K., Kwok P.C.L. (2011). Production methods for nanodrug particles using the bottom-up approach. Adv. Drug Deliv. Rev..

[262] He Y., Huang Y., Cheng Y. (2010). Structure evolution of curcumin nanoprecipitation from a micromixer. Cryst. Growth Des..

[263] Sudhakar B., NagaJyothi K., Ramana Murthy K. (2014). Nanosuspensions as a versatile carrier based drug delivery system–An overview. Curr. Drug Deliv..

[264] Kahraman E., Güngör S., Özsoy Y. (2017). Potential enhancement and targeting strategies of polymeric and lipid-based nanocarriers in dermal drug delivery. Ther. Deliv..

[265] Huang Y.-W., Cambre M., Lee H.-J. (2017). The toxicity of nanoparticles depends on multiple molecular and physicochemical mechanisms. Int. J. Mol. Sci..

[266] He C., Hu Y., Yin L., Tang C., Yin C. (2010). Effects of particle size and surface charge on cellular uptake and biodistribution of polymeric nanoparticles. Biomaterials.

[267] Jarvis M., Krishnan V., Mitragotri S. (2018). Nanocrystals: A perspective on translational research and clinical studies. Bioeng. Transl. Med..

[268] Petronella F., Pagliarulo A., Striccoli M., Calia A., Lettieri M., Colangiuli D., Curri M.L., Comparelli R. (2017). Colloidal nanocrystalline semiconductor materials as photocatalysts for environmental protection of architectural stone. Crystals.

[269] Lu Y., Li Y., Wu W. (2016). Injected nanocrystals for targeted drug delivery. Acta Pharm. Sin. B.

[270] Lee D.R., Park J.S., Bae I.H., Lee Y., Kim B.M. (2016). Liquid crystal nanoparticle formulation as an oral drug delivery system for liver-specific distribution. Int. J. Nanomed..

[271] Kobyliak N., Abenavoli L., Falalyeyeva T., Virchenko O., Natalia B., Beregova T., Bodnar P., Spivak M. (2016). Prevention of NAFLD development in rats with obesity via the improvement of pro/antioxidant state by cerium dioxide nanoparticles. Clujul. Med..

[272] Kobyliak N., Virchenko O., Falalyeyeva T., Kondro M., Beregova T., Bodnar P., Shcherbakov O., Bubnov R., Caprnda M., Delev D. (2017). Cerium dioxide nanoparticles possess anti-inflammatory properties in the conditions of the obesity-associated NAFLD in rats. Biomed. Pharmacother..

[273] Kobyliak N.M., Falalyeyeva T.M., Kuryk O.G., Beregova T.V., Bodnar P.M., Zholobak N.M., Shcherbakov O.B., Bubnov R.V., Spivak M.Y. (2015). Antioxidative effects of cerium dioxide nanoparticles ameliorate age-related male infertility: Optimistic results in rats and the review of clinical clues for integrative concept of men health and fertility. EPMA J..

[274] Pal Y., Deb P.K., Bandopadhyay S., Bandyopadhyay N., Tekade R.K., Tekade R.K. (2018). Chapter 3—Role of Physicochemical Parameters on Drug Absorption and Their Implications in Pharmaceutical Product Development. Dosage Form Design Considerations.

[275] Gorad R.S., Mandlik S.K., Gujar K.N. (2013). Liver Specific Drug Targeting Strategies: A Review. Int. J. Pharma. Sci. Res..

[276] Ghasemiyeh P., Mohammadi-Samani S. (2018). Solid lipid nanoparticles and nanostructured lipid carriers as novel drug delivery systems: Applications, advantages and disadvantages. Res. Pharm. Sci..

[277] Li M., Zhang W., Wang B., Gao Y., Song Z., Zheng Q.C. (2016). Ligand-based targeted therapy: A novel strategy for hepatocellular carcinoma. Int. J. Nanomed..

[278] Cunha S., Costa C.P., Moreira J.N., Sousa Lobo J.M., Silva A.C. (2020). Using the quality by design (QbD) approach to optimize formulations of lipid nanoparticles and nanoemulsions: A review. Nanomedicine.

[279] Kanthamneni N., Valiveti S., Patel M., Xia H., Tseng Y.-C. (2016). Enhanced bioavailability of danazol nanosuspensions by wet milling and high-pressure homogenization. Int. J. Pharm. Investig..

[280] Wang Y., Zhang L., Wang Q., Zhang D. (2014). Recent advances in the nanotechnology-based drug delivery of Silybin. J. Biomed. Nanotechnol..

[281] Ding Z., Xiao J., Zhang Y., Jiang Y., Chen W., Hu J., Guo Y., Zhang B. (2019). Pharmacokinetics and liver uptake of three Schisandra lignans in rats after oral administration of liposome encapsulating β-cyclodextrin inclusion compound of Schisandra extract. J. Liposome Res..

[282] Yan T., Yan N., Wang P., Xia Y., Hao H., Wang G., Gonzalez F.J. (2020). Herbal drug discovery for the treatment of nonalcoholic fatty liver disease. Acta Pharm. Sin. B.

[283] Musolino V., Gliozzi M., Scarano F., Bosco F., Scicchitano M., Nucera S., Carresi C., Ruga S., Zito M.C., Maiuolo J. (2020). Bergamot Polyphenols Improve Dyslipidemia and Pathophysiological Features in a Mouse Model of Non-Alcoholic Fatty Liver Disease. Sci. Rep..

[284] Granato D., Barba F.J., Kovačević D.B., Lorenzo J.M., Cruz A.G., Putnik P. (2020). Functional Foods: Product Development, Technological Trends, Efficacy Testing, and Safety. Annu. Rev. Food Sci. Technol..

[285] Khoo W.Y., Chrisfield B.J., Sae-tan S., Lambert J.D. (2020). Mitigation of nonalcoholic fatty liver disease in high-fat-fed mice by the combination of decaffeinated green tea extract and voluntary exercise. J. Nutr. Biochem..

[286] Saha P., Talukdar A.D., Nath R., Sarker S.D., Nahar L., Sahu J., Choudhury M.D. (2019). Role of Natural Phenolics in Hepatoprotection: A Mechanistic Review and Analysis of Regulatory Network of Associated Genes. Front. Pharmacol..

[287] Liu B., Zhang J., Sun P., Yi R., Han X., Zhao X. (2019). Raw Bowl Tea (Tuocha) Polyphenol Prevention of Nonalcoholic Fatty Liver Disease by Regulating Intestinal Function in Mice. Biomolecules.

[288] Kennedy D.O. (2014). Polyphenols and the human brain: Plant “secondary metabolite” ecologic roles and endogenous signaling functions drive benefits. Adv. Nutr..

[289] Liu H., Qiu N., Ding H., Yao R. (2008). Polyphenols contents and antioxidant capacity of 68 Chinese herbals suitable for medical or food uses. Food Res. Int..

[290] Zhou Y., Zheng J., Li Y., Xu D.-P., Li S., Chen Y.-M., Li H.-B. (2016). Natural Polyphenols for Prevention and Treatment of Cancer. Nutrients.

[291] Zhang Y., Rauf Khan A., Fu M., Zhai Y., Ji J., Bobrovskaya L., Zhai G. (2019). Advances in curcumin-loaded nanopreparations: Improving bioavailability and overcoming inherent drawbacks. J. Drug Target.

[292] Dogaru G., Bulboaca A.E., Gheban D., Boarescu P.M., Rus V., Festila D., Sitar-taut A.-V., Stanescu I. (2020). Effect of Liposomal Curcumin on Acetaminophen Hepatotoxicity by Down-regulation of Oxidative Stress and Matrix Metalloproteinases. In Vivo.

[293] Rezaei S., Sasani M.R., Akhlaghi M., Kohanmoo A. (2020). Flaxseed oil in the context of a weight loss programme ameliorates fatty liver grade in patients with non-alcoholic fatty liver disease: A randomised double-blind controlled trial. Br. J. Nutr..

[294] Bagherniya M., Nobili V., Blesso C.N., Sahebkar A. (2018). Medicinal plants and bioactive natural compounds in the treatment of non-alcoholic fatty liver disease: A clinical review. Pharmacol. Res..

[295] Beji R.S., Khemir S., Wannes W.A., Ayari K., Ksouri R. (2018). Antidiabetic, antihyperlipidemic and antioxidant influences of the spice cinnamon (Cinnamomum zeylanicumon) in experimental rats. Braz. J. Pharm. Sci..

[296] Hodges J.K., Sasaki G.Y., Bruno R.S. (2020). Anti-inflammatory activities of green tea catechins along the gut–liver axis in nonalcoholic fatty liver disease: Lessons learned from preclinical and human studies. J. Nutr. Biochem..

[297] Liu H., Zhong H., Leng L., Jiang Z. (2017). Effects of soy isoflavone on hepatic steatosis in high fat-induced rats. J. Clin. Biochem. Nutr..

[298] Yan T., Wang H., Cao L., Wang Q., Takahashi S., Yagai T., Li G., Krausz K.W., Wang G., Gonzalez F.J. (2018). Glycyrrhizin Alleviates Nonalcoholic Steatohepatitis via Modulating Bile Acids and Meta-Inflammation. Drug Metab. Dispos..

[299] Xu Y., Guo W., Zhang C., Chen F., Tan H.Y., Li S., Wang N., Feng Y. (2020). Herbal Medicine in the Treatment of Non-Alcoholic Fatty Liver Diseases-Efficacy, Action Mechanism, and Clinical Application. Front. Pharmacol..

[300] Bunnoy A., Saenphet K., Lumyong S., Saenphet S., Chomdej S. (2015). Monascus purpureus-fermented Thai glutinous rice reduces blood and hepatic cholesterol and hepatic steatosis concentrations in diet-induced hypercholesterolemic rats. BMC Complement Altern. Med..

[301] Zhu B., Qi F., Wu J., Yin G., Hua J., Zhang Q., Qin L. (2019). Red Yeast Rice: A Systematic Review of the Traditional Uses, Chemistry, Pharmacology, and Quality Control of an Important Chinese Folk Medicine. Front. Pharmacol..

[302] Zhu M., Li M., Zhou W., Yang Y., Li F., Zhang L., Ji G. (2019). Qianggan extract improved nonalcoholic steatohepatitis by modulating lncRNA/circRNA immune ceRNA networks. BMC Complement Altern. Med..

[303] Ebrahimi-Mameghani M., Aliashrafi S., Javadzadeh Y., Asghari Jafarabadi M. (2014). The Effect of Chlorella vulgaris Supplementation on Liver En-zymes, Serum Glucose and Lipid Profile in Patients with Non-Alcoholic Fatty Liver Disease. Health Promot. Perspect..

[304] Fan J.-G., Shanghai Multicenter Clinical Cooperative Group of Danning Pian Trial (2004). Evaluating the efficacy and safety of Danning Pian in the short-term treatment of patients with non-alcoholic fatty liver disease: A multicenter clinical trial. HBPD INT.

[305] Lou S.Y., Liu Y., Ma Y.Y., Chen H.Y., Chen W.H., Ying J., He Y.M., Wang W.J. (2008). Effects of Yiqi Sanju Formula on non-alcoholic fatty liver disease: A randomized controlled trial. Chin. J. Integr. Med..

[306] Guo H., Zhong R., Liu Y., Jiang X., Tang X., Li Z., Xia M., Ling W. (2014). Effects of bayberry juice on inflammatory and apoptotic markers in young adults with features of non-alcoholic fatty liver disease. Nutrition.

[307] Rodriguez-Ramiro I., Vauzour D., Minihane A. (2016). Polyphenols and non-alcoholic fatty liver disease: Impact and mechanisms. Proc. Nutr. Soc..

[308] Gundermann K.-J., Gundermann S., Drozdzik M., Mohan Prasad V.G. (2016). Essential phospholipids in fatty liver: A scientific update. Clin. Exp. Gastroenterol..

[309] Panyod S., Wu W.-K., Ho C.-T., Lu K.-H., Liu C.-T., Yung-lin C., Lai Y.-S., Chen W.-C., Lin Y.-E., Lin S.-H. (2016). Diet Supplementation with Allicin Protects against Alcoholic Fatty Liver Disease in Mice by Improving Anti-Inflammation and Antioxidative Functions. J. Agric. Food Chem..

[310] Seif el-Din S., Sabra A.-N., Hammam O., Ebeid F., El-Lakkany N. (2014). Pharmacological and Antioxidant Actions of Garlic and—Or Onion in Non Alcoholic Fatty Liver Disease (Nafld) in Rats. J. Egypt. Soc. Parasitol..

[311] Li H., Li Q., Liu Z., Yang K., Chen Z., Cheng Q., Wu L. (2017). The Versatile Effects of Dihydromyricetin in Health. Evid. Based Complement Altern. Med..

[312] Sen S., Chakraborty R., Gracia-Sancho J., Salvadó J. (2017). Chapter 19—Herbs, Gastrointestinal Protection, and Oxidative Stress. Gastrointestinal Tissue.

[313] Faran S.A., Asghar S., Khalid S.H., Khan I.U., Asif M., Khalid I., Farooq Gohar U., Hussain T. (2019). Hepatoprotective and Renoprotective Properties of Lovastatin-Loaded Ginger and Garlic Oil Nanoemulsomes: Insights into Serum Biological Parameters. Medicina.

[314] Bahadori E., Farjami Z., Rezayi M., Lngari H., Darroudi M., Avan A., Ghayour-Mobarhan M. (2019). Recent advances in nanotechnology for the treatment of metabolic syndrome. Diabetes Metab. Syndr..

[315] Mousavi Z., Hassanpourezatti M., Najafizadeh P., Rezagholian S., Rhamanifar M.S., Nosrati N. (2016). Effects of subcutaneous injection MnO2 micro-and nanoparticles on blood glucose level and lipid profile in rat. Iran. J. Med. Sci..

[316] Jia J., Li F., Zhou H., Bai Y., Liu S., Jiang Y., Jiang G., Yan B. (2017). Oral Exposure to Silver Nanoparticles or Silver Ions May Aggravate Fatty Liver Disease in Overweight Mice. Environ. Sci. Technol..

